# Impact of biochar, compost, and sludge amendments on the soil water balance of a sandy soil

**DOI:** 10.1007/s42773-025-00509-4

**Published:** 2026-01-19

**Authors:** Slaven Tenodi, Snežana Maletić, Marijana Kragulj Isakovski, Jens Kruse, Lutz Weihermüller

**Affiliations:** 1 Department of Chemistry, Biochemistry and Environmental Protection, University of Novi Sad Faculty of Sciences, Trg Dositeja Obradovića 3, Novi Sad, 21000 Republic of Serbia; 2 Institute of Bio- and Geosciences-IBG-3, Agrosphere Institute, Forschungszentrum Jülich GmbH, Leo-Brandt-Strasse, 52425 Jülich, Germany

**Keywords:** Sandy soil amendments, Water retention, Lysimeter experiment, Soil hydraulic properties, Biochar, Compost

## Abstract

Sandy soils, with inherently low water retention and poor hydraulic properties, present significant challenges for sustainable agriculture, particularly in water-limited conditions. This study investigates the impact of biochar, sludge, and compost amendments on the soil hydraulic properties and water balance of a sandy soil. A 441-day lysimeter experiment evaluated six treatments: biochar (A), sludge (B), compost (C), biochar + sludge (D), biochar + compost (E), and biochar + sludge + compost (F). Results showed that combined treatments outperformed single amendments, with treatment F (biochar + sludge + compost) exhibiting the most pronounced improvements in soil water dynamics. This treatment reduced cumulative drainage by over 40% relative to individual amendments and exhibited higher average soil water content and more stable water storage across seasonal fluctuations. Biochar addition enhanced soil porosity and water-holding capacity, while compost and sludge improved retention through organic matter input and fine particle contributions. Treatments containing biochar reduced drainage and increased actual evaporation, indicating improved soil water retention and availability. Saturated hydraulic conductivity, field capacity, and plant available water were closely correlated with observed drainage behavior, confirming the functional relevance of these soil hydraulic indicators. Statistical analyses, including one-way ANOVA and Tukey’s HSD, supported the significance of treatment differences in drainage and actual evaporation. Overall, the study demonstrates that integrating biochar, compost, and sludge can synergistically enhance water retention, reduce drainage, and stabilize soil water contents in sandy soils. These findings offer practical insights for improving water use efficiency and resilience in arid and semi-arid agroecosystems.

**Supplementary Information:**

The online version contains supplementary material available at 10.1007/s42773-025-00509-4.

## Highlights

- Biochar + sludge + compost (F) reduced drainage by over 40%, optimizing water retention in sandy soils.
- Biochar-inclusive treatments minimized evaporation losses, improving soil water availability.
- Synergistic amendments enhanced soil structure and water balance, aiding sustainable agriculture in dry regions.

**Supplementary Information:**

The online version contains supplementary material available at 10.1007/s42773-025-00509-4.

## Introduction

Climate change presents a global challenge, impacting agricultural systems and food production (IPCC 2014). Additionally, world population will surpass 9 billion by 2050, and consequently, food production needs to increase by 70 − 85% to maintain global food, fiber, and fodder demand (Dhankher and Foyer 2018). Given the scarcity of arable land in Europe, utilizing marginal or previously underused land has become a strategy to sustain or increase agricultural production (Schröder et al. 2022). Unfortunately, the use of marginal sandy soils, characterized by low water retention and nutrient availability, presents a significant challenge for agricultural practices and water management (Zhang et al. 2016). Sandy soils are characterized by low organic matter content, poor water retention, and high hydraulic conductivity, conditions exacerbated by climate change, making them unsuitable for high-yield agriculture, particularly during droughts (Villagra-Mendoza and Horn 2018). To address these limitations, various organic amendments such as biochar, sludge, and compost have been proposed to potentially improve the soil hydraulic characteristics (Castellini et al. 2015; Lim et al. 2016).

Biochar, a product of the pyrolysis of organic matter, has gained attention as a promising soil amendment, particularly for sandy and coarse-textured soils. Its ability to enhance soil water retention and mitigate drought impacts makes it especially relevant in the context of climate variability and increasing drought frequency. Studies by Li et al. (2021) and Khan et al. (2024) have demonstrated biochar’s efficacy in improving soil hydraulic characteristics by increasing field capacity, plant-available water content, and microporosity while stabilizing soil water content dynamics. These properties highlight its value in improving porosity and water retention in coarse-textured soils, addressing key agricultural and environmental challenges.

However, existing literature on biochar's influence on soil water-related properties often remains fragmented. Many studies focus on isolated properties under controlled laboratory conditions (Zhang et al. 2016; Stylianou et al. 2020), overlooking the broader interactions in dynamic field environments. For instance, Zhang et al. (2016) evaluated the saturated hydraulic conductivity (*K*_*s*_) and actual evaporation in sandy soils, but did not assess the overall water balance or plant-available water. Similarly, Lim et al. (2016) and Ouyang et al. (2013) provided important insights into *K*_*s*_ and retention characteristics, respectively. Yet, the interaction under real-world conditions with variable rainfall and potential evaporation remains insufficiently addressed. This gap highlights the need for integrative, long-term field studies that capture the complexity of amendment-soil–water dynamics. Table 1 provides a comprehensive summary of studies assessing biochar's impacts on soil hydraulic and physical properties. While it compiles key data across various experimental conditions, it intent to underscore the diversity of approaches and outcomes reported in literature, rather than suggest consistency or predictability. Notably, the listed parameters (e.g., bulk density, WHC, *K*_*s*_, porosity) serve as proxies for broader soil functioning, and the variations observed reflect not only differences in biochar type and soil texture, but also methodological differences across studies. Although micropores in biochar have been associated with improved water retention, the term “smaller pores” must be used with greater specificity. For instance, micropores (< 2 µm) retain water more tightly, contributing to water held at pressure heads larger than permanent wilting point (PWP), while mesopores (2–50 µm) are more relevant for plant-available water and fast drainage. Some studies (Liang et al. 2021) suggest that mesopore development is more important, for increasing field capacity, and thus, more relevant for agronomic water use. The previous phrasing that biochar with "smaller pores retains water more effectively as the coarse soil will do" is therefore imprecise and potentially misleading as coarse soils inherently drain quickly due to dominance of macropores. Therefore, those soils cannot be used as a benchmark for improved retention. Biochar modifies this by adding micro- and mesopores to the soil, thus enabling the retention of water that would otherwise drain through sandy soil profiles.

**Table 1:** Summary of biochar studies on soil hydraulic and physical properties

Literature	Soil type	Biochar type	Feedstock	Biochar rate	Measured characteristics
Villagra-Mendoza and Horn (2018)	Sandy soil	Coarse biochar	Unspecified	2.5 to 5%	BD, WHC, Porosity, Retention, Pore distribution
Zhang et al. (2016)	Sandy soil	Large pore biochar	Unspecified	1 to 10%	Evaporation rate, Hydraulic conductivity
Castellini et al. (2015)	Clay soil	General biochar	Fruit tree pruning	5 to 30%	*K*_*s*_, Field capacity, Saturation
Igalavithana et al. (2017)	Sandy loam	Corn residue biochar	Corn residue	Up to 10%	BD, WHC, *K*_*s*_, Retention
Ouyang et al. (2013)	Sandy loam	Dairy manure biochar	Dairy manure	50%	Aggregate stability, Water retention curve
Edeh et al. (2020)	Various	Various types	Various feedstocks	< 30 to > 200 t ha^−1^	AWC, Field capacity, *K*_*s*_, WHC
Rabbi et al. (2021)	Various	Review (various)	Various	Variable	BD, WHC, *K*_*s*_, Saturation, Pore size
Stylianou et al. (2020)	Loamy sand	Coffee-derived biochar	Coffee waste	Variable	*K*_*s*_, Saturation, Water content
Zhou et al. (2018)	Sandy loam	Maize cob biochar	Maize cob	High dose	Porosity, WHC, *K*_*s*_, Soil moisture
Chen et al. (2023)	Silty sand	Peanut shell biochar	Peanut shell	High dose	WHC, Permeability, Water retention curve
Dokoohaki et al. (2018)	Cropland soil	Fine particle biochar	Unspecified	10 t ha^−1^	WHC, *K*_*s*_, Soil moisture dynamics
Lim et al. (2016)	Various	Wood and plant biochar	Hardwood, pine	1 to 5%	*K*_*s*_
Liu et al. (2022)	Sandy soil	Maize straw biochar	Maize straw	5%	BD, TP, Field capacity, *K*_*s*_
Abel et al. (2013)	Sandy soil	Maize silage and beechwood biochar	Maize silage, beechwood	1 to 5%	BD, WHC, Water repellency
Mao et al. (2019)	Sandy soil	Various feedstocks	27 feedstocks	Variable	Hydrophobicity, WHC, Water repellency
Wiersma et al. (2020)	Sandy soil	Rice husk biochar	Rice husk	10 t ha^−1^	Water retention, Hydrophobicity

BD = bulk density; WHC = water holding capacity; TP = total porosity; *K*_*s*_ = saturated hydraulic conductivity

Biochar’s role in altering soil water retention curves has been also demonstrated in a few studies, yet findings remain variable. For example, Ouyang et al. (2013) reported that dairy-manure-derived biochar increased saturated water content and decreased residual water content in sandy loam and silty clay soils, indicating altered pore architecture. Similarly, Edeh et al. (2020) observed improvements in field capacity and available water content across various soil types but noted that the magnitude of effects varied by biochar feedstock and rate of application. This heterogeneity reflects a critical knowledge gap: how specific combinations of biochar properties and soil types interact under realistic field conditions to influence overall soil hydraulic functioning.

To date, relatively few studies have evaluated biochar within the context of full water balance assessments, which integrate actual evaporation, drainage, and storage. Long-term lysimeter experiments, like the one presented in this study, are particularly suited for this purpose. As emphasized by Villagra-Mendoza and Horn (2018) and Zhou et al. (2018), field conditions introduce dynamic variables such as precipitation variability, temperature fluctuation, and biological activity that cannot be replicated in laboratory columns. Thus, findings from such controlled environments, while valuable, offer only partial insights into the performance of soil amendment.

While biochar has drawn substantial research attention, compost and sludge also offer potential to improve soil water retention. Compost is widely recognized for enhancing organic matter content and structure, particularly by increasing macroporosity and water-holding capacity through aggregation (Rivier et al. 2022). In sandy soils, compost has been shown to buffer against water stress and improve pore size distribution, which can lead to increased field capacity and plant water availability (Whelan et al. 2013; Zemánek 2011). However, some studies also report diminishing effects over time, particularly in heavier soils, where the compost decomposes and its structural benefits decline (Castellini et al. 2022). Sludge, although less frequently discussed in the context of soil water retention, has been found to significantly increase fine fractions and organic content, thus improving retention in coarse-textured soils (Głąb et al. 2018). Its inclusion in amendment strategies is further supported by findings from Saudy et al. (2021), who demonstrated improved pore distribution and water availability in faba bean fields treated with spot-applied sludge. The sludge’s high clay and humic content, in particular, contributes to microporosity that retains water at less negative matric potentials, i.e., near saturation.

Despite the potential benefits of each amendment, studies examining combinations of biochar with compost or sludge are rare. Research by Ali et al. (2024) and El-Bially et al. (2023) highlights that integrating multiple organic materials can produce synergistic effects. For instance, Ali et al. (2024) found that combining compost or vermicompost with bio-stimulants enhanced plant growth and substrate water holding capacity more than single amendments. El-Bially et al. (2023) similarly demonstrated that biochar in combination with mycorrhiza improved both plant yield and soil resilience. These studies reinforce the rationale for testing co-applications, especially in soils where multiple limitations (e.g., low organic matter, poor soil structure, or low water retention) coexist.

Moreover, the selection of amendment rates in this study reflects both practical considerations and findings from prior research. A relatively low rate of biochar (1% w/w) was selected to test efficacy without compromising feasibility or risking soil saturation effects, while higher doses of compost (5%) and sludge (20%) reflect typical agronomic practices and build on ratios used in field trials by Saudy et al. (2021) and Saudy et al. (2023). The goal was to test not only individual amendment performance but also their interactions under realistic application scenarios.

The novelty of this study lies in its integrative approach. Unlike many prior investigations that focused on single properties or short-term experiments, this study evaluates soil hydraulic properties and water balance over 441 days using a lysimeter setup. This allows for simultaneous monitoring of soil water content, drainage, and actual evaporation across treatments. Furthermore, the inclusion of both individual and combined amendments enables assessment of potential synergistic or antagonistic effects, which remain underexplored in current literature.

This study also aims to fill a crucial gap by testing whether biochar’s water-retention benefits can be enhanced or modulated by co-applying it with compost or sludge. The hypothesis is that combining amendments with distinct physical and chemical properties can create more stable and effective pore networks, leading to improved water retention, reduced drainage, and enhanced plant-available water. By comparing single and combined treatments across key water balance parameters, the study provides a comprehensive perspective on the role of organic amendments in sustainable soil management.

In summary, while biochar, compost, and sludge have each demonstrated potential to improve soil hydraulic functioning, significant knowledge gaps remain regarding their interactive effects and performance under field conditions. This study addresses these gaps by employing a long-term lysimeter experiment to evaluate amendment impacts on water balance components in a marginal sandy soil. The findings aim to inform best practices for soil amendment strategies, especially in regions facing water scarcity and soil degradation.

## Materials and methods

### Lysimeter setup

**Fig. 1:**
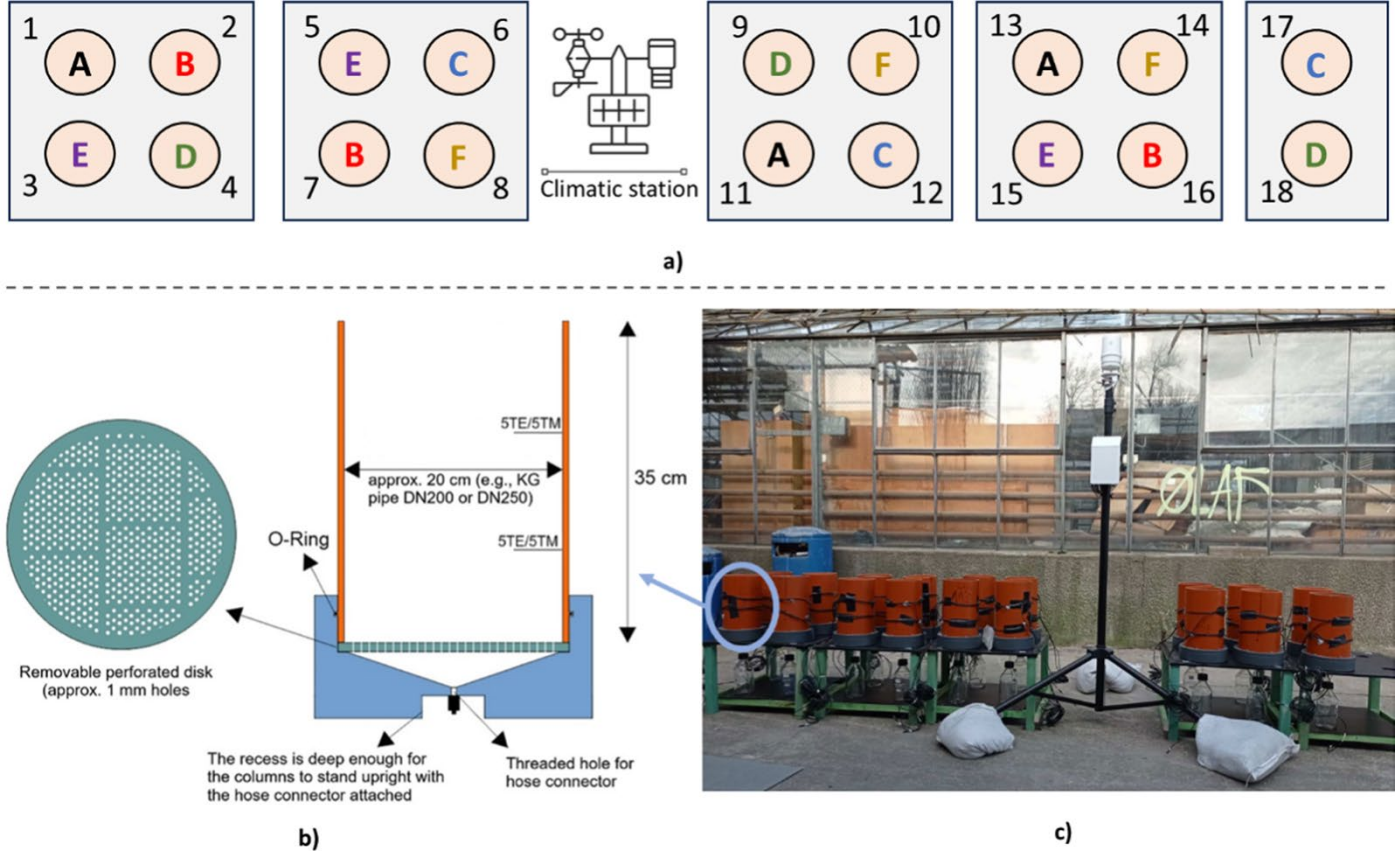
Experimental setup for the lysimeter study. **a** Top panel indicates the randomized setup of the individual treatments with treatment: sandy soil + biochar (treatment A), sandy soil + sludge (treatment B), sandy soil + compost (treatment C), sandy soil + biochar + sludge (treatment C), sandy soil + biochar + compost (treatment E), and sandy soil + biochar + sludge + compost (treatment F). **b** Schematic of a single lysimeter, indicating PVC column, coarse sand drainage layer, 5TE/5TM sensors at 10 and 20 cm depth, and drainage outlet. **c** Photograph of the lysimeter setup, with one lysimeter circled and connected to its corresponding schematic representation in panel (**b**)

The lysimeter experiment was conducted at the University of Novi Sad, Faculty of Science (45°14′42.30"N, 19°51′13.12"E), within a monitored, fenced area. The study utilized 18 lysimeters, each constructed from PVC pipes with a height of 35 cm, an outer diameter of 20 cm, and an inner diameter of 18.28 cm. The bottom of each lysimeter was fitted with a removable perforated plastic disk with 1 mm holes (see Fig. 1). A 3-cm layer of coarse sand was placed at the base of each lysimeter to facilitate drainage while preventing soil loss. The lysimeters filled with soil mixed with different organic amendments (treatment A to F, see description below) were placed randomly in the lysimeter facility. SWC and soil temperature within each lysimeter were measured using 5TE and 5TM sensors (Meter Group, Munich, Germany) installed 10 and 20 cm from the bottom of the lysimeters. Sensors were connected to EM50 or ZL6 data loggers (Meter Group, Munich, Germany) for continuous data recording. Data were downloaded weekly and performance was checked to ensure data integrity.

Drainage (leachate) from each lysimeter was directed through small hoses connected to the lower boundary of the lysimeters into 1 L precleaned glass sampling bottles with polypropylene caps and PTFE/silicone septa. The septa allowed for secure hose connections and facilitated leachate collection. Each bottle was pre-labelled and weighed to ensure accurate mass measurement of collected leachate. Drainage mass was measured on a laboratory scale with a range up to 3200 g and a resolution of 0.01 g. Drainage collection frequency was based on visual inspection of the lysimeter drainage collection bottles. Sampling was triggered whenever the accumulated drainage reached a measurable level (typically ≥ 50 mL) to ensure accurate volume determination. To avoid potential overflow or data loss during forecasted larger rainfall events, drainage bottles were checked and emptied in advance when heavy precipitation was expected. Based on the measured drainage mass, the drainage volume was calculated and related to surface area and converted to mm equivalent relative to the surface area.

### Soil and soil amendments

The lysimeters were filled with a marginal sandy soil taken from a site close to the Danube River near the drinking water source “Petrovaradinska ada” in Novi Sad, Serbia (45°15′39.89ʺN, 19°51′55.08ʺE). The sandy soil was characterized by 65 ± 4.6% sand (2000–50 µm), 16 ± 6.4% silt, and 10 ± 3.6% clay and had a low soil organic carbon (C_org_) content of 0.24 ± 0.05%. To evaluate the impact of single and combined applications of commonly used soil amendments on soil hydraulic properties, the sandy soil was mixed with compost, biochar, or sludge as well as their combinations. This resulted in six different treatments (replicated three times, *N* = 3), each with specific amendment proportions: treatment A—sandy soil + 1% (w/w) biochar, treatment B—sandy soil + 20% (w/w) sludge, treatment C—sandy soil + 5% (w/w) compost, treatment D—sandy soil + 1% (w/w) biochar + 20% (w/w) sludge, treatment E—sandy soil + 1% (w/w) biochar + 5% (w/w) compost, and treatment F—sandy soil + 1% (w/w) biochar + 20% (w/w) sludge + 5% (w/w) compost. The selected amendment rates were chosen to reflect commonly reported application ranges in the literature while balancing material characteristics and their expected functional effects on soil hydraulic properties. Biochar was applied at a relatively low rate (1% w/w) to improve soil structure and porosity without excessive alteration of soil bulk density, while higher proportions of sludge (20% w/w) and compost (5% w/w) were used to enhance organic matter content and water retention.

Treatment B (sandy soil + sludge) was chosen to represent a finer-textured baseline soil compared to the initial sandy soil. By incorporating sludge, the mixture provides improved water retention properties and this soil acts as a second reference point for evaluating the different soil amendments involving biochar and compost.

The biochar used in this study was produced from *Miscanthus* feedstock through slow pyrolysis at 550 °C at the Technical University Aachen (RWTH), Germany. A 100 g subsample was analyzed for particle size distribution by dry sieving using a 2 mm mesh. The results showed that approximately 74% of the mass consisted of particles > 2 mm, while 26% was < 2 mm. The largest biochar fragments reached up to ~ 3 cm in length, though most coarse particles were spherical with a diameter of approximately 5 mm. This relatively coarse particle structure is representative of typical field-grade biochar and may influence soil pore size distribution and water retention behavior. The addition of the C-rich (77.2%) biochar aims to improve soil structure, increase nutrient retention, and reduce the leaching potential of contaminants in the amended soils. After mixing the sandy soil with biochar (1%), the resulting soil organic carbon (C_org_) content was calculated to be approximately 1.01% for the mixtures. For the sandy soil mixture with the sludge (treatment B, D, and F), a clay rich sludge dredged from the Begej Channel, Serbia was mixed with the sand. The overall mixture of sand and sludge (treatment B) resulted in a soil containing 64.1% sand (2–50 µm), 14.5% silt, and 14.3% clay and a C_org_ of 0.68%.

The compost used in this study was derived from green waste sourced from Novi Sad, Serbia. This organic material was incorporated into the sandy soil for treatments C, E, and F. Although specific measurements of organic carbon content were not available, the compost amendment is known to improve soil structure, increase C_org_, enhance nutrient availability, and increase water retention capacity in the amended soils. The physico-chemical characteristics of the raw materials used are listed in Table 2. Granulometric composition was determined only for the sandy soil and sludge, and organic carbon content measurements were available for sandy soil, sludge, and biochar; therefore, only these values are reported in Tables 2 and 3.

**Table 2:** Physico-chemical properties of raw materials used in the experiment

Material and mixture	Feedstock/origin	Grain size distribution	C_org_ (%)
Sandy soil	Danube River bank, “Petrovaradinska ada” Novi Sad, Serbia	Sand: 65 ± 4.6%	0.24 ± 0.05
		(2000–50 µm)	
		Silt: 16 ± 6.4%	
		Clay: 10 ± 3.6%	
Biochar	*Miscanthus* feedstock (pyrolysis at 550°C), Technical University Aachen (RWTH), Germany	–	77.2
Sludge	Sludge from Begej channel near Novi Sad, Serbia	Sand: 60.4 ± 6.22%	2.45 ± 0.63
		(2000–50 µm)	
		Silt: 8.3 ± 2.9%	
		Clay: 31.3 ± 6.3%	
Compost	Green waste sourced from Novi Sad, Serbia	–	–

**Table 3:** Effect of soil amendments on treatment composition and hydraulic properties

Property type	Sandy soil + biochar (treatment A)	Sandy soil + sludge (treatment B)	Sandy soil + compost (treatment C)	Sandy soil + biochar + sludge (treatment D)	Sandy soil + biochar + compost (treatment E)	Sandy soil + biochar + sludge + compost (treatment F)
Composition of treatments						
Granulometric composition	–	Sand: 64.1% Silt: 14.5% Clay: 14.3%	–	–	–	–
C_org_ (%)	1.01	0.68	–	1.45	–	–
Soil hydraulic properties and bimodal Durner parameters						
BD (g cm^−3^)	1.39	1.38	1.35	1.38	1.37	1.26
* K*_*s*_ (cm day^−1^)	664.13 ± 31.11	109.44 ± 2.78	864.29 ± 31.58	53.71 ± 3.08	518.69 ± 3.27	31.62 ± 3.27
* θ*_*s*_ (cm^3^ cm^−3^)	0.401	0.425	0.405	0.436	0.413	0.465
* θ*_*r*_ (cm^3^ cm^−3^)	0.004	< 0.001	0.018	0.002	0.039	< 0.001
* α*_1_ (cm^−3^)	0.0228	0.0189	0.0134	0.0214	0.0171	0.0158
* α*_2_ (cm^−3^)	0.0283	0.0276	0.0291	0.0241	0.0260	0.0223
* n*_1_	1.484	1.281	1.732	1.308	1.877	1.330
* n*_2_	11.081	7.809	14.670	9.140	12.425	7.305
* w*	0.605	0.399	0.613	0.293	0.581	0.197
* λ*	1.687	0.307	3.251	0.529	-0.329	0.460
Water contents (cm^3^ cm^−3^) at given pressure (cm) selected at field capacity (FC)						
FC @ 100	0.152	0.131	0.175	0.096	0.156	0.073
FC @ 200	0.116	0.113	0.125	0.081	0.110	0.060
FC @ 250	0.105	0.107	0.111	0.076	0.098	0.056
* h* = 15,848 (permanent wilting point at pF 4.2)	0.018	0.034	0.023	0.023	0.041	0.015
Plant available water (cm^3^ cm^−3^) for given field capacities @ –100, –200, and –250 cm						
100–15,848	0.134	0.097	0.152	0.073	0.116	0.058
200–15,848	0.098	0.078	0.103	0.058	0.045	0.069
250–15,848	0.087	0.072	0.089	0.053	0.057	0.041

All soil amendments were dried and thoroughly mixed with the dried sandy soil using a construction mixer. The mixtures were then filled into the lysimeters with stepwise compaction to ensure a uniform bulk density of approximately 1.4 g cm^−3^ across depths and treatments. During the experiment over 441 days the soil was kept bare, and when needed any upcoming vegetation such as herbs and moss was removed.

### Climatic data and irrigation

**Fig. 2:**
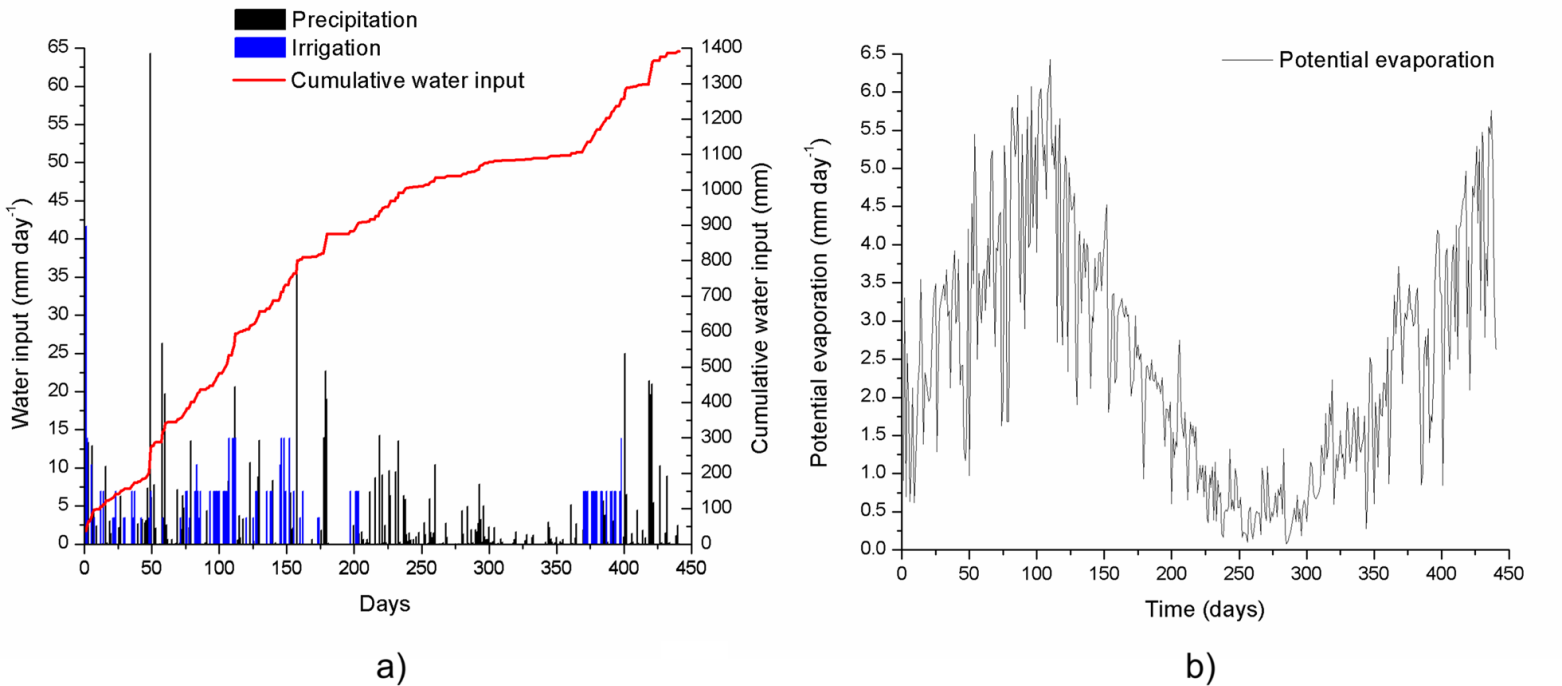
**a** Daily water input (mm day^−1^) via precipitation and irrigation, alongside the cumulative water input (mm) during the 441-day experimental period and **b** potential evaporation (E_pot_) (mm). In both plots the days of the experiment are shown, and the start of the experiment was on 31st of March 2023

ATMOS 41 all-in-one weather station (Meter Group, Munich, Germany) recorded hourly climate data (precipitation, relative humidity, wind speed, air temperature, and solar radiation), stored via a ZL6 data logger (Meter Group, Munich, Germany). Data were downloaded weekly and performance was checked to ensure data integrity. The hourly potential evaporation (E_pot_) was calculated according to FAO56 (Allen et al. 1998) and subsequently aggregated to daily values as also done for the measured SWCs (see Fig. 2b). To ensure high drainage during the experiment, especially in summer and dry periods, all treatments received additional irrigation. In total, 126 irrigation events were performed over the experimental period, whereby irrigation was applied using a perforated disk to evenly distribute the water across the soil surface and to avoid hard splash of the added water. This method prevented surface ponding and ensured uniform water infiltration. Irrigation was applied simultaneously across all lysimeters, using the same method, and at the same time for each irrigation event, ensuring consistent water distribution and eliminating variability in irrigation application between treatments. As a result, the total water intake, comprising both precipitation and irrigation, was identical for all lysimeters throughout the experiment. Both irrigation and precipitation (water intake) are presented graphically in Fig. 2a to illustrate cumulative water input across the experimental period.

### Laboratory measurements of soil hydraulic properties

Soil hydraulic properties were analyzed at the Institute of Bio- and Geosciences (Aerosphere, IBG-3), Forschungszentrum Jülich GmbH, using standardized laboratory methods. For this, 250 cm^3^ cylinders were filled with the soil-mixtures to the same BD as used in the lysimeters. After filling the cylinders, the soil was gradually saturated from the bottom to ensure complete saturation. The saturated hydraulic conductivity *K*_*s*_ was measured using a permeameter with the falling-head method (KSAT Device, Meter Group, Munich) (Dane and Topp 2002), whereby the same sample was measured three times and the arithmetic mean was calculated.

Soil hydraulic characteristics (water retention and hydraulic conductivity characteristics) were determined by the evaporation method using the HYPROP® system (Meter Group, München, Germany) as described by Schindler et al. (2010) in combination with the WP4® Dewpoint Potentiometer (Decagon Devices, WA, USA). Two different soil hydraulic models describing the retention and hydraulic conductivity functions were fitted to the HYPROP® data, namely the unimodal van Genuchten (van Genuchten 1980) model and, second the dual-porosity Durner (1994) model, using the HYPROP Fit software (Meter Group, Munich, Germany). The retention function for the dual-porosity Durner model can be written as:

1 $$\theta (h)={\theta }_{r}+{(\theta }_{s}-{\theta }_{r})\sum_{i=1}^{k}{\omega }_{i}{Se}_{i}$$

with

2 $${Se}_{i}={\left[1+{\left|{\alpha }_{i}h\right|}^{{n}_{i}}\right]}^{{-m}_{i}},$$

where *θ*_*r*_ and *θ*_*s*_ are the residual and the saturated water contents [cm^3^ cm^−3^], respectively, *k* is the order of porosity in the soil system (here *k* = 1 for the unimodal (van Genuchten) and *k* = 2 for dual-porosity model), *Se* is the effective saturation [−], *ω*_*i*_ is the weighting factor (∑*ω*_*i*_ = 1). *α*_*i*_ [cm^−1^], *n*_*i*_ [−], and *m*_*i*_ [−] are empirical parameters, whereby *α*_*i*_ can be related to the inverse of the air entry values and *n*_*i*_ to the width of the pore size distribution, whereas *m*_*i*_ is classically related to *n*_*i*_ by *m*_*i*_ = 1–1/*n*_*i*_. *h* is the pressure head [cm].

The relative soil hydraulic conductivity function *K(h)* is given by Priesack and Durner (2006):

3 $$K(h)={{{K}_{s}\sum_{i=1}^{k}{\omega }_{i}{Se}_{i}}^{\lambda }}{\left[\frac{\sum_{i=1}^{k}{\left(1-\left(1-{Se}_{i}^{1/{m}_{i}}\right)\right)}^{{m}_{i}}}{\sum_{i=1}^{k}{\omega }_{i}{\alpha }_{i}}\right]}^{2}$$

where *K*_*s*_ is the saturated hydraulic conductivity [cm day^−1^], which was kept fixed during the fitting of the soil hydraulic model to the measured data.

Based on the knowledge of the retention characteristics the plant available water (PAW) was derived as the difference between field capacity (FC) and permanent wilting point (PWP). Therefore, water contents at different pressure heads (*h* = −100, −200, and –250 cm) were calculated and used to approximate field capacity (FC) under progressively decreasing matric potentials, following common conventions in soil physics (e.g., Romano and Santini 2002; Tuller and Or 2004). These approximations reflect near-saturated conditions typical of sandy soils where FC is often estimated at pressure heads between −100 and –300 cm. Water content as PWP was calculated at pF = 4.2 (*h* = −15,849 cm) and the differences between the FC and PWP was assigned as PAW.

### Statistical analysis

Statistical analysis was performed in Origin 8.0 (OriginLab) using one-way ANOVA to compare water fluxes (storage, drainage, and calculated actual evaporation (E_act_)) across treatments. The analysis was conducted at a significance level of *p* = 0.05. Following the ANOVA, Tukey’s post-hoc test was applied to identify significant differences between treatment groups. To visualize differences in water fluxes, box plots were generated using Origin 8.0.

## Results and discussion

### Soil hydraulic properties under amendment treatments

Fitting the two soil hydraulic models (Eqs. 1 and 3) to the measured data revealed distinct dual porosity in the mid-pressure head range and all treatments were better described by the dual-porosity Durner model. The dual-porosity character of most soils has already been demonstrated by Zhang et al. (2022), and the addition of soil amendments to a fairly coarse soil is likely to enhance this characteristic further. The measured soil hydraulic characteristics and the fitted dual-porosity (Durner) model are depicted in Supplementary Material Figure SM1 and the estimated soil hydraulic parameters are listed in Table 3. The results obtained reveal differences in hydraulic properties between soils treated with individual amendments (biochar, sludge, and compost) and their combinations. In general, the impact of BD after amendment is difficult to evaluate, as the BDs used in the laboratory were the same as those used in the lysimeters and largely depended on the ability to densely pack the material into the lysimeters. Therefore, findings such as those reported by Liang et al. (2021), which demonstrated biochar’s ability to decrease BD due to its porous structure and low particle density, cannot be directly confirmed in our study. On the other hand, the impact of the compost amendment is visible as adding compost reduced BD within a narrow range, and for the co-amendment with biochar and sludge, the BD even dropped substantially. It is known that compost can contribute to BD reduction, as noted by Głąb et al. (2018), where sewage sludge and compost combinations significantly decreased BD by diluting the denser mineral fraction. Treatment F showed enhanced effects that exceeded those of single amendments, suggesting beneficial interactions among the materials, with greater reductions in BD than with single amendments, likely due to enhanced aggregation and structural stabilization from organic matter inputs.

Most differences between the treatments can be detected in the measured *K*_*s*_, with the highest values observed in soils amended with compost and/or biochar but without sludge. Biochar has been shown to influence both water flow and retention by modifying pore architecture, facilitating greater water movement (Zhou et al. 2018). Similar effects on soil macrostructure and saturated hydraulic conductivity (*K*_*s*_) have been observed with compost amendments, which enhance water retention, hydraulic conductivity, and soil aggregation, as supported by studies such as Whelan et al. (2013), Rivier et al. (2022), and Aggelides and Londra (2000). However, the role of sludge points in the opposite direction, with smaller *K*_*s*_ for all sludge-amended soils. Most likely, the fine-textured sludge filled the coarse pores between the sand, reducing total cross-sectional area and generating smaller pores in which the water is transported slower (Abu-Sharar 1993). The combination of biochar and sludge showed smaller *K*_*s*_ values than the sludge amendment alone and the combination of sludge, compost, and biochar even showed the smallest *K*_*s*_ values over all treatments, even at low bulk density of these soils. Although, the exact mechanisms are unclear, it is likely that particle size distribution played a role. Finer particles from sludge and compost may have filled larger pores, thereby blocking larger pores and likely also reducing pore connectivity. Therefore, this could be a result of single or combined effects, including the formation of smaller pores due to particle size distribution (Liu et al. 2016), the impact of organic materials on soil packing (Villagra-Mendoza and Horn 2018), the combination of amendments creating complex pore networks (Yan et al. 2021), and surface interaction effects (Ajayi et al. 2016).

Measured FC showed, that the amendment of the sandy soil with biochar (treatment A) and compost (treatment C) increased FC compared to the amendment with sludge for all FC calculated (FC @ –100, –200, and –250), whereby the differences between the sludge and the biochar/compost amendment is larger for the FC @ –100 (16 and 33% improvement) compared to the FC @ –250 (−2 and 3% difference), indicating that most changes in the retention characteristics due to the amendment are here in the close saturation part of the retention curve and that the curves are closer to each other at smaller pressure heads (here *h* = − 250 cm). On the other hand, co-amendment of biochar with sludge (treatment D) or the triple amendment (treatment F) reduced the FC over all pressure heads compared to the single compost and biochar amendment and lowest FCs were found for the triple amended soil with a reduced water content at FC of 41% to 53% of the water contents at same FC for the biochar- and compost-amended soil, respectively. The higher observed FC for the biochar and compost amended soils (treatment A and C) aligns with findings by Edeh et al. (2020), who reported that the amendment with biochar increases FC and PAW by redistributing pore sizes toward micropores that retain plant-accessible water. Compost, on the other hand, can add organic colloids to the soil system that enhance WHC by increasing microporosity, as supported by Al-Omran et al. (2019). The amendment with biochar or compost seems to not only increase FC but as discussed also *K*_*s*_, and therefore, the macropore regions (improving *K*_*s*_) and micropores (enhancing FC) are impacted. The results also highlight the effectiveness of sludge in increasing FC, even if the FC calculated were slightly less than those for the biochar or compost amended soil. The observed increase in field capacity (FC) following sludge amendment can be attributed to its fine texture and organic matter content, which enhance microporosity and promote the formation of water-retaining microaggregates—a mechanism supported by previous findings on compost and sludge amendments reported by Głąb et al. (2018), Al-Omran et al. (2019), and Rivier et al. (2022). The reason for the decrease in FC in the triple-amended soil is somehow unclear as the sludge reduced FC, as can be seen for the sludge amended soil (treatment C) (water contents for all FCs calculated > 0.076 cm^3^ cm^−3^), but the positive effects of the biochar and compost seem not to be present as the water contents for all FCs for treatment F are calculated < 0.073 cm^3^ cm^−3^ (see Table 3).

The water contents at permanent wilting point were highest for the biochar and compost amendment (treatment E with 0.041 cm^3^ cm^−3^) and smallest for the triple amendment (biochar + compost + sludge−treatment F with 0.015 cm^3^ cm^−3^). The sludge amendment also showed relatively high water contents at PWP with 0.034 cm^3^ cm^−3^, whereas the other amendments varied between 0.018 (treatment A) and 0.023 cm^3^ cm^−3^ (treatment C and D). Liang et al. (2021) reported that biochar can increase water contents at PWP, which they attributed to the biochar’s ability to retain tightly bound water within its internal (fine) pores. As there is no reference (no amendment) in the setup used in this study, the effect of the biochar on PWP cannot be finally judged, but compared to the same amount of compost added (treatment C), no outperforming of the biochar can be detected, and the water contents at PWP are even slightly smaller than those calculated for the compost. The impact of the sludge on the water contents at PWP is much higher, but here, we have to keep in mind that the sludge was added at a higher percentage than the biochar or compost. Nevertheless, Głąb et al. (2018) pointed out that sludge, as well as compost amendments, can increase the water contents at PWP, due to the formation of microaggregates that hold water more effectively.

Interestingly, the combined application of biochar and compost resulted in the highest water content at permanent wilting point (PWP) among all treatments, indicating their synergistic effect on retaining tightly bound water. On the other hand, compost and biochar amendment along with the sludge reduced the water contents at FC, for which the reasons are not fully clear.

Finally, we evaluated the calculated plant-available water (PAW). In general, the highest PAW was found for all FC calculated for the biochar (treatment A) and compost amendment (treatment C), which are also characterized by the highest water contents at all FCs and also by low water contents at PWP (0.019 cm^3^ cm^−3^). On the other hand, the lowest PAW was found for the triple amendment (treatment F), followed by the combined biochar and compost (treatment E), and the biochar sludge amendment (treatment D). Treatments D and F were also characterized by low water contents at FCs, while treatment E with the biochar and compost showed high to intermediate water contents at FC but also the highest water content at PWP, and therefore, only low PAW. A strong correlation was found between *K*_*s*_ and FC at *h* = −100 cm (*R*^2^ = 0.81), indicating that the amendments similarly influenced mesopore development. The correlation between *K*_*s*_ and water contents at FC measured at *h* = −250 cm also showed a positive trend with a slightly lower *R*^2^ of 0.51. Surprisingly, also the PAW is correlated to measured *K*_*s*_ (*R*^2^ = 0.92), even though the water at PWP is not related to the pores itself and more to the film water surrounding the matrix. But looking at the correlation between water contents at FC calculated at *h* = −100 cm and PAW calculated for the same pressure head at FC one will find a strong correlation with higher PAW for higher FC with an *R*^2^ of 0.94.

These findings show that the amendments influenced single-point soil characteristics in distinct ways. While individual treatments had clear trends, combined amendments sometimes counteracted or altered the effects seen in the single applications. It should be noted that the soil hydraulic parameters presented in Table 3, including FC and PAW, were derived from fitted soil water retention models based on laboratory measurements. Due to the model-based nature of these data and the lack of replicates across treatments, statistical testing (e.g., ANOVA) was not applicable. As an alternative for quantitative comparison of treatment effects on water retention under field conditions, statistical analyses of cumulative storage are presented in Sect. 3.2.4. Nevertheless, the results indicate that the selection and combination of the soil amendments should be based on specific goals, such as improving infiltration by increasing, for example, *K*_*s*_, enhancing water retention, or optimizing plant-available water. The synergistic effects observed in the combination treatments highlight the potential for tailored amendment strategies to maximize the performance of sandy soils. Future research should explore long-term impacts and the effects of varying amendment ratios on hydraulic properties, as well as their interactions with soil texture and climatic conditions.

### Lysimeter experiments

Because single-point soil water characteristics alone do not fully describe soil functioning after amendment, we conducted a 441-day lysimeter experiment to assess the effects of biochar, sludge, and compost and analyzed the hydraulic responses in a sandy soil. By continuously monitoring SWC, soil temperature, and drainage, and combining these measurements with climatic data, the study captured a comprehensive view of water dynamics under real-world conditions.

Soil temperature was measured using sensors positioned at 10 and 20 cm from the bottom of the lysimeters to monitor thermal dynamics across the treatments (see Supplementary material). Although treatment differences were minimal, clear seasonal trends emerged, with higher soil temperatures in summer and lower values in winter—consistent with expected climatic variation. Temperature fluctuations were slightly greater at 10 than at 20 cm depth, likely due to closer exposure to atmospheric conditions. Importantly, soil temperature did not differ significantly between treatments, reducing the likelihood of temperature-driven variability in upper boundary water fluxes.

#### Water input and potential evaporation

Figure 2a illustrates the daily water input (mm), differentiated between natural precipitation (black bars) and irrigation (blue bars), along with the cumulative water input over the 441-day experiment (red line). As can be seen, natural precipitation was variable, with some extreme high rainfall events exceeding 20 mm on days 49, 58, 112, 136, 179, and 421 after the start of the experiment (31st of March 2023). It is evident that no clear seasonal pattern emerged in the precipitation distribution. The overall cumulative natural precipitation over the 441 days of the experiment summed up to 790 mm. To ensure that each lysimeter received enough water to generate sufficient drainage, the lysimeters were additionally irrigated with up to 14 mm day^−1^ (first irrigation was done on dry mixtures adding up to 42 mm throughout a day). In total 126 irrigations were performed, summing up to 604 mm. Summing up natural precipitation and irrigation yielded 1394 mm over the course of the study period.

Figure 2b shows the daily sums of calculated potential evaporation (E_pot_), which followed a clear seasonal pattern. E_pot_ peaked at up to 5.5 mm day^−1^ during late spring and summer, and was much lower in late fall, winter, and early spring. In total, E_pot_ summed up to 1090 mm over the 441 days of the experiment indicating that the water balance was positive with more incoming water (precipitation + irrigation) compared to potential loss via E_pot_.

#### Soil water content dynamics across treatments

**Fig. 3:**
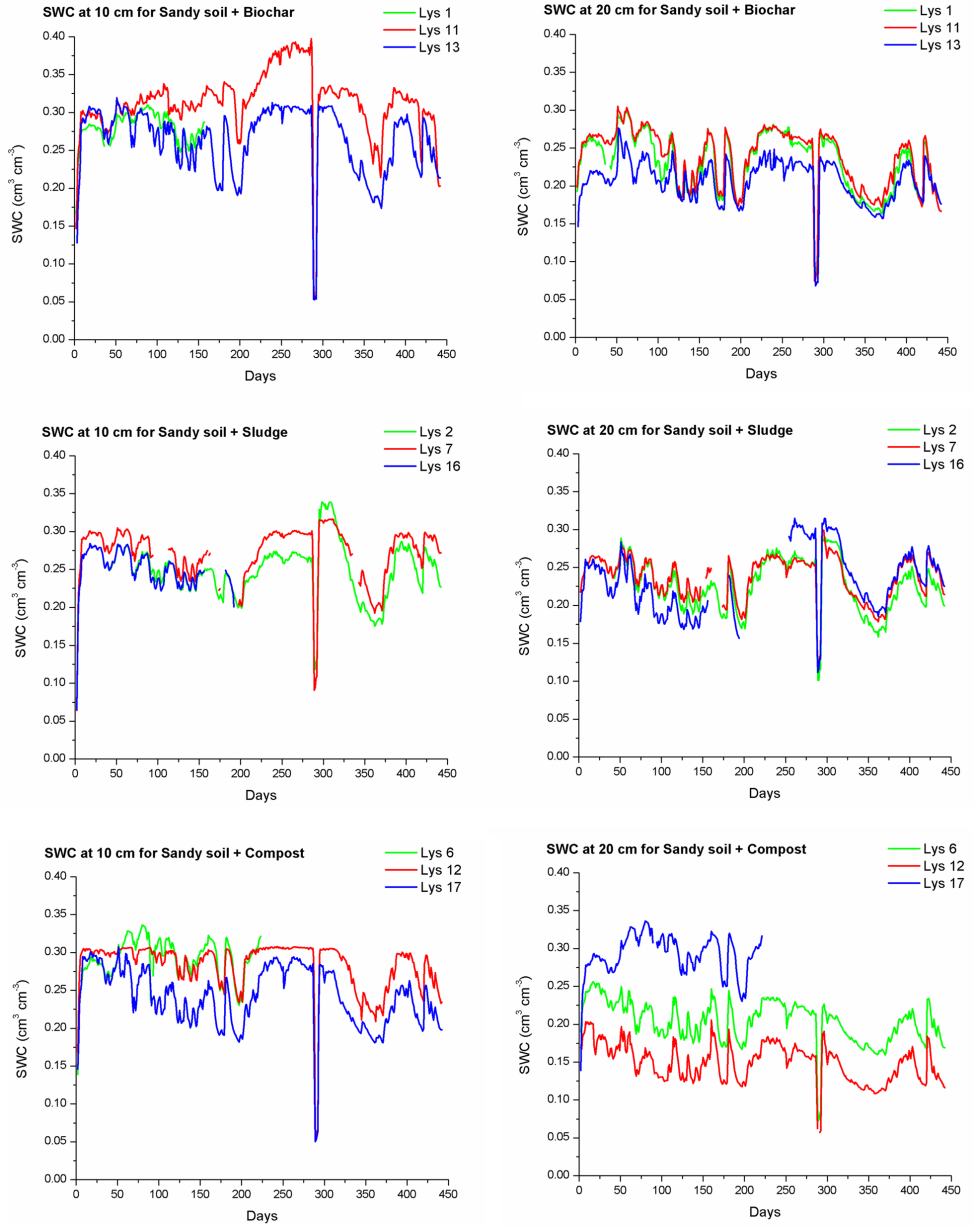
Soil water content (SWC) (cm^3^ cm^−3^) over the experimental period of 441 days for different soil treatments: sandy soil + biochar (treatment A), sandy soil + sludge (treatment B), and sandy soil + compost (treatment C), at two depths (10 and 20 cm from the bottom of the lysimeters) for the 3 replicated lysimeters. Each subplot corresponds to one soil treatment with three lysimeter replicates (*N* = 3), shown as individual colored lines. Days of the experiment are shown, and the start of the experiment was at 31st of March 2023

**Fig. 4:**
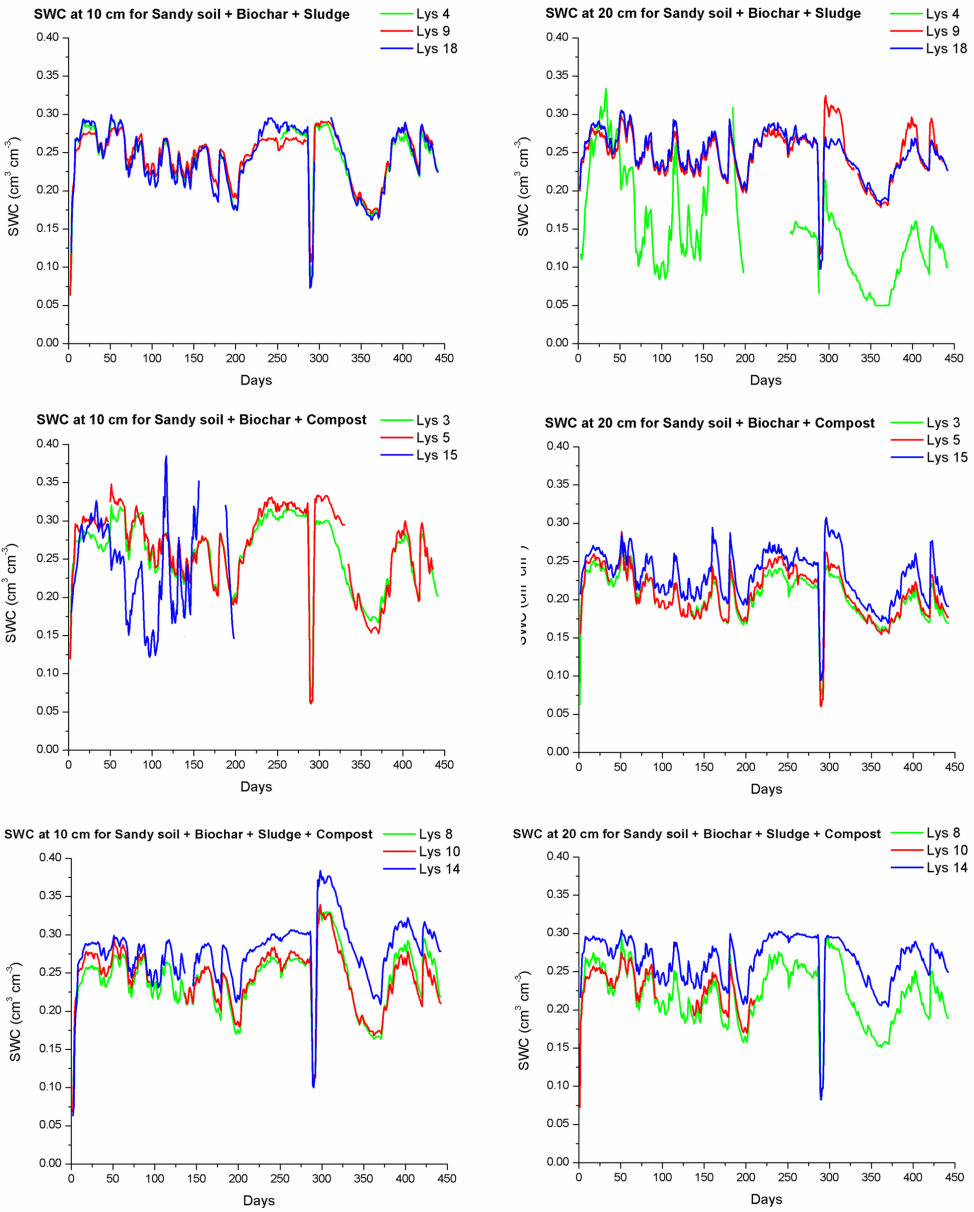
Soil water content (SWC) (cm^3^ cm^−3^) over the experimental period of 441 days for different soil treatments: sandy soil + biochar + sludge (treatment D), sandy soil + biochar + compost (treatment E), and sandy soil + biochar + sludge + compost (treatment F), at two depths (10 and 20 cm from the bottom of the lysimeters) for the 3 replicated lysimeters. Each subplot corresponds to one soil treatment with three lysimeter replicates (*N* = 3), shown as individual colored lines. Days of the experiment are shown, and the start of the experiment was at 31st of March 2023

The SWC measurements across treatments are presented in Fig. 3 (for treatments A–C) and Fig. 4 (for treatments D–E), revealing distinct patterns related to the type and combination of amendments applied, offering insights into the amendments' effectiveness in sandy soils. Here it has to be noted that some sensors failed to record readings intermittently due to logger or sensor malfunctions. Loggers were changed after breakdown as quick as possible, but sensors could not be changed as they were buried entirely in the lysimeters. Biochar, sludge, and compost are known to enhance soil water retention by altering pore structure and increasing water holding capacity (WHC). In this study, biochar alone (treatment A) led to higher initial SWC values than sludge (treatment B), supporting previous findings that biochar improves water retention by increasing porosity and reducing bulk density (Villagra-Mendoza and Horn 2018). Among the combined treatments, biochar + sludge and biochar + compost generally showed higher and more stable SWC levels, while treatment B exhibited intermediate values reflecting the baseline properties of improved soil texture after adding sludge to the sandy soil. The stability in SWC for these combined treatments may be attributed to the complementary properties of biochar’s porosity and the organic content in sludge or compost. Organic amendments like sludge (semi-organic and semi-clayic) and compost not only contribute to enhanced soil structure but may enhance soil water content buffering by forming a more stable organic matrix under variable conditions. This finding is consistent with those of Rivier et al. (2022), who reported that compost enhances water retention and plant water use efficiency by improving soil structure and pore distribution.

Furthermore, biochar + sludge + compost (treatment F) demonstrated the highest average SWC values over time, indicating that the combined effects of these three amendments created a cumulative benefit for water retention. This treatment’s ability to retain water may be particularly advantageous in sandy soils prone to rapid drainage, as it could reduce the frequency of irrigation. Notably, SWC in the triple-amended soil remained relatively stable over time, even under conditions that typically accelerate drainage, reflecting the amendment's potential for improved soil water stability, which is critical for sustaining plant growth.

In contrast, treatments with biochar alone (A and D) showed slightly greater variability in SWC, particularly at the 10-cm depth. This indicates that while biochar increases WHC, its effects may be less stable when used alone compared to combinations with other organic amendments. This variability highlights the role of organic amendments in buffering SWC against fluctuations, a finding that resonates with studies such as that by Castellini et al. (2022), who observed that compost alone can enhance water retention but even more effectively if co-applied with biochar.

**Fig. 5:**
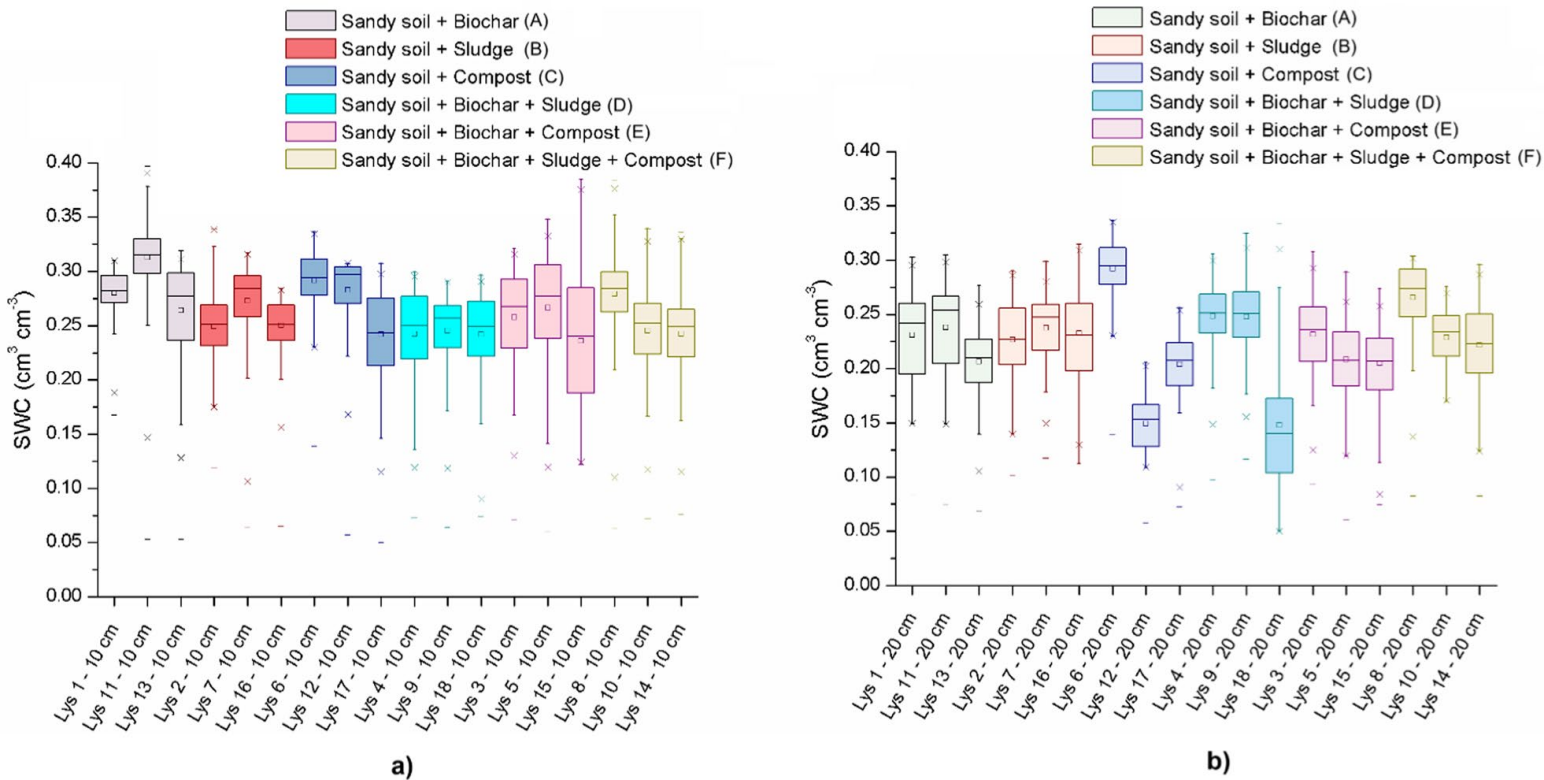
Box plots for soil water content (SWC) for the different treatments over the 441-day experimental period for: **a** measurements at 10 cm from the bottom and **b** measurements at the 20 cm from the bottom of the lysimeters. Treatments show differing central tendencies and variabilities in SWC, with the highest medians observed in treatments D (biochar + sludge) and F (biochar + sludge + compost). Although, trends were visible, one-way ANOVA did not detect statistically significant differences among treatments (*p* > 0.05), likely due to high intra-treatment variability (see Supplementary Table SM2 for full statistical results)

It is important to note that given the continuous and autocorrelated nature of the daily SWC measurements, formal statistical testing (e.g., ANOVA) was not applied directly to the time series. Instead, SWC variability was summarized using boxplots for each treatment as presented Fig. 5 to allow for robust visual comparison of treatment effects. Complementary statistical analyses were performed on the cumulative water storage values derived from SWC (see Sect. 3.2.4), enabling quantitative assessment of amendment impacts on water retention. The central line within each box represents the median SWC value for the treatment. The box itself illustrates the interquartile range, which captures the middle 50% of the data. Whiskers extend to the minimum and maximum values within 1.5 times the interquartile range, while individual stars or points outside this range denote statistical outliers. The box plots also illustrate central tendencies and stability across treatments, with notable differences between SWC values measured at 10-cm and 20-cm depths.

The median SWC values, represented by the lines within each box, reveal the central tendency of water retention for each treatment, while the box height and whiskers indicate variability, offering insights into how consistently each treatment retains water.

Measurements at 10 cm (see Fig. 5a) from the bottom of the lysimeters generally show higher SWC values with less variability across treatments compared to those measured at 20 cm (see Fig. 5b), likely due to gravitational water accumulation near the bottom of the lysimeters. The combined treatments, particularly biochar + sludge (treatment D) and biochar + compost (treatment E), showed stable and relatively high SWC values at 10 cm. This stability suggests that biochar, when combined with organic amendments like sludge and compost, retains soil water more effectively in the lower part of the lysimeter, which could serve as a critical water reserve accessible to plants during dry periods.

In contrast, measurements at 20 cm from the bottom of the lysimeters exhibit more variability and generally lower median SWC values across treatments, as water accumulation at this level is less pronounced. Here, treatments with biochar alone show wider interquartile ranges, indicating more variability in SWC. This suggests that while biochar enhances soil water retention, its effects may cause larger SWC fluctuation in the lysimeters when used alone. This observation aligns with that of Zhang et al. (2016), who found that biochar alone exhibited variable water retention, particularly under fluctuating atmospheric conditions.

The most complex treatment F (biochar + sludge + compost), however, demonstrates the most stable and consistent SWC values across both measurement depths, with a narrow interquartile range and high median SWC at 10 and 20 cm. This suggests a cumulative effect of the triple amendment, where biochar’s porosity and the organic content of sludge and compost contribute to a balanced soil structure and wide pore size distribution that uniformly retains soil water throughout the soil profile. This uniformity across both sensor levels could support plant root development across the soil profile as it could provide consistent water source for plant growth, and therefore, reducing the dependency on frequent irrigation.

#### Impact of amendments on drainage

**Fig. 6:**
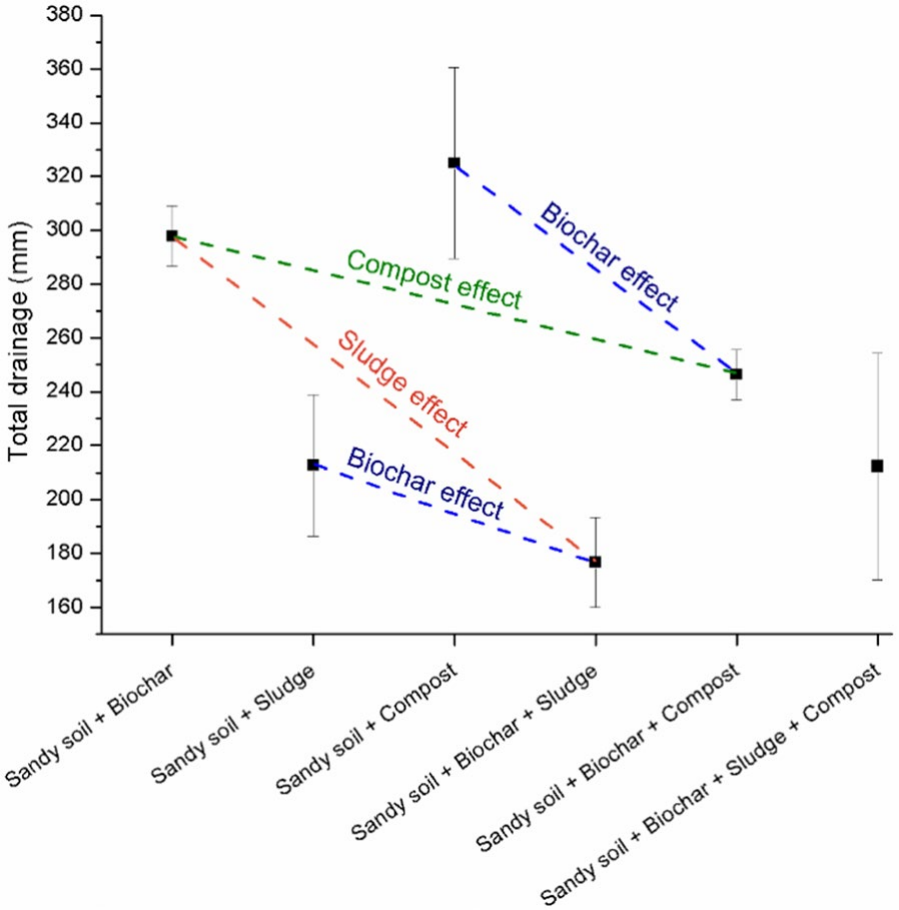
Cumulative measured drainage (mm) after 441 days of the experimental period for the different soil treatments (*n* = 3 for each treatment). Statistical analysis (ANOVA and Tukey HSD) showed that treatment F (biochar + sludge + compost) significantly reduced drainage compared to treatments A (biochar only) and C (compost only) (*p* < 0.01) (see Supplementary material, Table SM1)

Figure 6 shows the total measured drainage sampled at the bottom of the lysimeters. The data show that biochar, sludge, and compost had differing impacts on total drainage. Biochar-containing treatments (A, D, E, and F) reduced cumulative drainage, as indicated by the blue dashed line. This suggests improved water retention and possibly greater evaporative losses in sandy soils.

The "Sludge effect" (red dashed line) also shows a reduction in drainage and assumes higher actual evaporation when sludge was added, especially in combination with biochar (treatment D). The “Compost effect” (green dashed line) demonstrates a moderate reduction in drainage, though its impact varied depending on the combination with other amendments (treatment E and F). These findings provide insight into how specific soil amendments influence not only the soil hydraulic properties but also the soil functioning in terms of water storage, drainage, and actual evaporation, which are all essential for optimizing soil treatments in sandy environments.

**Fig. 7:**
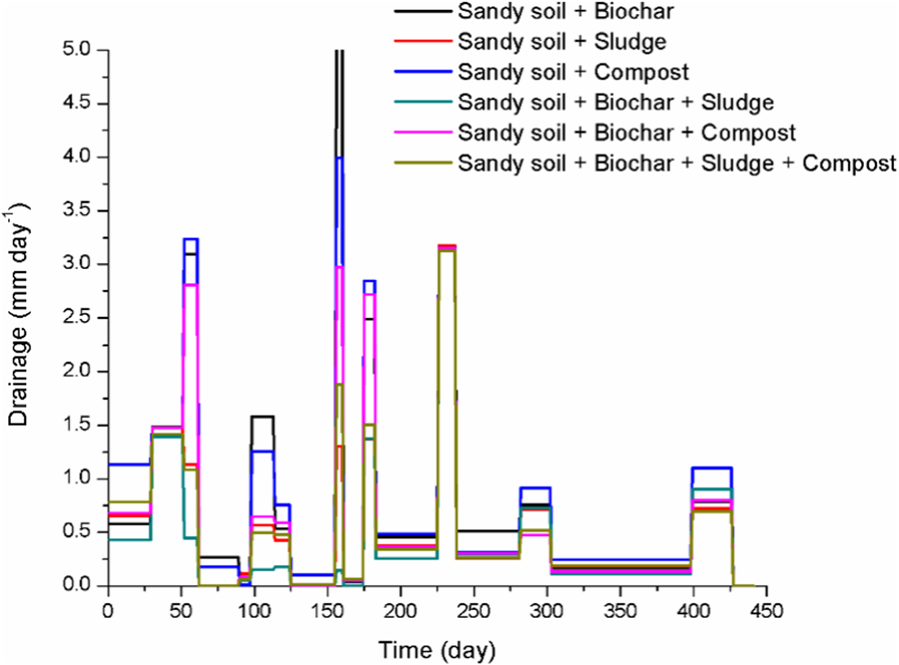
Average daily drainage (mm day^−1^) of the different sampling periods for the different soil treatments (*n* = 3 for soil treatments). Significant differences among treatments were identified via Tukey HSD post-hoc test (*p* < 0.05), with treatment F showing the lowest drainage rates (see Supplementary material, Table SM1)

Drainage measurements presented in Fig. 7 revealed variations across treatments, reflecting the differing impacts of the amendments on water movement through the soil profile. Treatments involving biochar, particularly when combined with sludge and compost (treatments D, E, and F), consistently exhibited lower cumulative drainage compared to treatments with sludge or compost alone. For instance, the treatment combining biochar, sludge, and compost (treatment F) displayed the lowest overall drainage, demonstrating the cumulative benefits of combining amendments to reduce water loss and improve soil water retention. In contrast, the sandy soil + sludge treatment (treatment B) and the compost-only treatment (treatment C) had comparatively higher drainage, indicative of their limited ability to retain water when not combined with biochar.

Between treatments, the porous structure of biochar likely improved water retention and limited percolation, contributing to reduced drainage (Li et al. 2021). Sludge, with its higher clay content, further stabilized water retention when combined with biochar, as seen in treatments D and F. Compost alone moderately reduced drainage, likely due to its organic matter content improving water-holding capacity as reported by Rivier et al. (2022). However, its effect was stronger when combined with biochar and sludge. These complementary effects were most pronounced in treatment F, where the triple combination produced the lowest drainage values and the most stable water retention. This is consistent with findings in this study, which demonstrate the importance of combining amendments to optimize water dynamics in sandy soils.

The observed differences among treatments also underline the potential trade-offs between water retention and drainage reduction. While compost improves soil structure and enhances SWC buffering, biochar provides long-term stability in SWC by improving soil porosity and reducing rapid water percolation. Li et al. (2021) emphasize the long-term potential of biochar to enhance soil water retention under varying climatic conditions. The synergistic use of biochar with other amendments, as seen in our study, could further enhance water retention and resilience in sandy soils. The complementary nature of these amendments highlights the need for tailored application strategies to achieve specific water management goals in sandy soils.

The drainage data reveal periodic spikes, particularly on days 49 and 179, which align with the most intense precipitation events observed in Fig. 2a. While these spikes suggest the possibility of some overflow in the sampling bottles during extreme rainfall, any such overflow is likely minimal and would not significantly impact the accuracy of the recorded drainage volumes. Efforts were made to minimize the risk of overflow, and any minor incidents that may have occurred would not alter the conclusions or observations drawn from the study. Despite these potential uncertainties, the drainage data trends remain robust and provide valuable insights into the effects of the amendments on water retention and drainage under varying environmental conditions.

#### Water balance

**Fig. 8:**
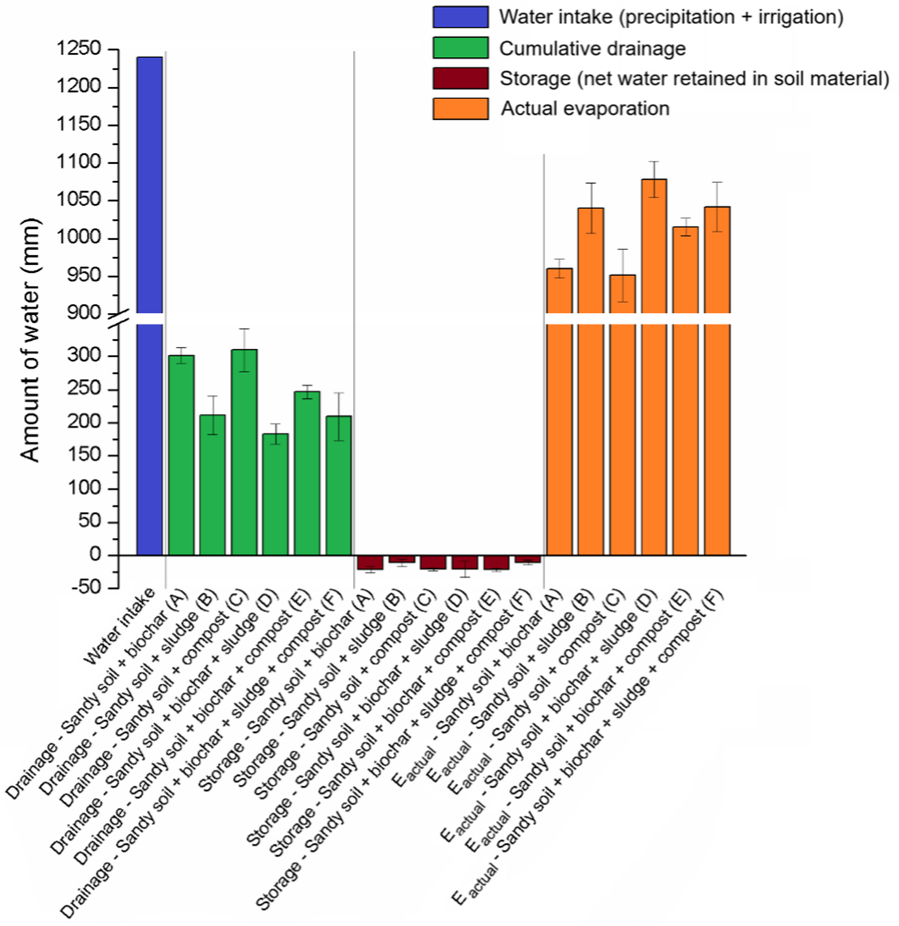
Water balance components over the 441-day experimental period, including water intake (precipitation + irrigation), drainage, storage, and actual evaporation, for the different soil treatments: sandy soil + biochar (**A**), sandy soil + sludge (**B**), sandy soil + compost (**C**), sandy soil + biochar + sludge (**D**), sandy soil + biochar + compost (**E**), and sandy soil + biochar + sludge + compost (**F**). Statistical analysis (see Supplementary material, Sect. 2) showed significant differences among treatments for drainage and actual evaporation, while differences in water storage were not statistically significant

In a next step, the water balance for each lysimeter was calculated based on water inputs (precipitation and irrigation), soil water storage (derived from SWC measurements), and drainage. Actual evaporation was calculated by the missing water in the overall balance. Figure 8 presents the water balance analysis, illustrating how different soil amendments affected drainage, water storage, and actual evaporation. To further interpret the water balance results, statistical analysis using ANOVA and Tukey HSD was conducted to identify significant differences in drainage, storage (as a proxy for SWC), and actual evaporation among the treatments. Table 4 summarizes significant differences in drainage and actual evaporation across treatments, while storage data showed no significant variation. Table 4 presents only the most critical results from the ANOVA and Tukey HSD analyses, focusing on significant findings and their implications, whereas detailed statistical outputs, including all pairwise comparisons and ANOVA summaries, are available in the Supplementary material (see Tables SM1, SM2, and SM3). The ANOVA results revealed a statistically significant variation in cumulative drainage among treatments (F = 13.18, *p* = 0.0002), emphasizing the influence of organic amendments on water movement through the soil profile. Tukey HSD identified significant reductions in drainage for treatments involving biochar, particularly in combination with sludge or compost.

**Table 4:** Results of ANOVA and Tukey HSD tests showing significant pairwise comparisons for drainage, storage, and actual evaporation

Variable	ANOVA *F*-statistic	ANOVA *p*-value	Significant pairwise comparison (Tukey HSD)	Mean difference (range)	*p*-value (adjusted)
Drainage	13.18	0.0002	A vs C, A vs E, B vs D, B vs E, D vs F	− 126.84–98.59	0.0005–1.0000
Storage	2.31	0.1086	–	− 10.75–10.73	0.3070–1.0000
Actual evaporation	10.50	0.0005	A vs C, A vs E, B vs D, B vs F, C vs E, D vs E, D vs F, E vs F	− 88.74–126.94	0.0009–1.0000

For instance, treatment F (biochar + sludge + compost) consistently exhibited the lowest cumulative drainage. The mean differences between treatment F and others, such as treatment A (biochar alone) and treatment C (compost alone), show that combining amendments can yield greater water-savings than using each amendment alone. These findings align with the hydraulic properties of biochar and the structural enhancements provided by sludge and compost.

The storage term, representing SWC, is expressed as a negative value on Fig. 8 to represent water retained within the system, calculated as the difference between inputs and outputs in the water balance. These values exhibited variability across treatments but did not reach statistical significance in the ANOVA (*F* = 2.31, *p* = 0.1086). Despite the lack of significance, trends in the Tukey HSD analysis suggest that combined amendments, such as treatments D, E, and F, contributed to improved SWC stability. These trends support the observations of enhanced pore connectivity and organic carbon contributions from biochar and compost, as previously discussed. Cumulative actual evaporation data showed significant differences among treatments (*F* = 10.50, *p* = 0.0004). Tukey HSD highlighted notable pairwise differences, particularly between treatments lacking biochar and those with combined amendments. Treatment F demonstrated moderate evaporation rates, balancing water retention with atmospheric loss. This stability underscores the importance of integrating biochar with organic amendments to optimize soil water availability while mitigating evaporation losses.

Treatment B (sandy soil + sludge) also used as the control to analyze the impact of biochar and compost on this soil, represents a baseline soil mixture with improved texture compared to pure sandy soil but without the addition of biochar or compost. As can be seen, this treatment exhibited intermediate water storage and drainage, reflecting the contribution of sludge in enhancing water retention as can also be seen in the treatments where sludge was added with biochar (treatment D) and along with compost and biochar (treatment F). In contrast, all amendments without sludge (treatments A, C, and E) showed higher drainage slightly higher storage changes, proving that the water retention is lower compared to those soils amended with sludge. The influence of biochar is again evident in comparisons such as treatment B vs. D and treatment C vs. E, where the addition of biochar consistently reduced drainage. As the water storage was only affected minor, the actual evaporation calculated by the mass balance was also affected, with higher evaporation losses for the treatments where no biochar was added to the soil.

This is consistent with the known properties of biochar, which enhances soil porosity and water retention by impacting the pore size distribution, especially in increasing microporosity as stated by Khan et al. (2024). As previously concluded, the amendment of sludge in combination with compost or biochar complement each other, with biochar enhancing pore connectivity and also film water at lower pressure heads, while sludge improves soil structure with its semi-clay content, and compost providing additional organic matter to buffer SWC fluctuations. These findings are consistent with prior studies, such as those by Rivier et al. (2022), which highlighted compost’s ability to improve water retention and soil structure, and Villagra-Mendoza and Horn (2018), who demonstrated biochar’s enhancement of mesoporosity and overall soil hydraulic properties. Furthermore, findings reported by Castellini et al. (2022) support our findings, as they stated that co-applications of biochar and compost reduced drainage by improving pore structure and water retention, even though the changes in drainage between treatment B and F are only minor with also small changes in the storage term. The slightly lower evaporation loss for the triple treatment (F) compared to treatment D (sludge and biochar) aligns with research by Naeini and Cook (2000), who noted, that compost-based amendments reduce evaporation through insulation and SWC buffering.

Overall, the water balance analysis highlights the impact of biochar amendment. The addition of biochar to the soil amended with sludge (treatment B) showed less drainage and lower actual evaporation compared to the same soil additionally amended with biochar (treatment D) with slightly lower drainage and higher evaporation losses.

A clear correlation exists between soil hydraulic properties and system responses, such as drainage. For example, *K*_*s*_ is well correlated to drainage with higher drainage for higher *K*_*s*_ (*R*^2^ = 0.92). Same but with lower *R*^2^ of 0.66 holds for the field capacity measured at *h* = −100 cm and drainage, as well as for the correlation between PAW calculated for FC at *h* = −100 cm with an *R*^2^ of 0.83. Based on these regressions, it can be concluded that point soil hydraulic parameters, such as *K*_*s*_, FC, and PAW, are reliable indicators or predictors of soil functioning, particularly in terms of drainage.

Overall, the results highlight the potential of combining biochar with compost and sludge to improve the hydraulic performance of sandy soils. Such strategies offer practical solutions for improving water management and supporting sustainable agricultural practices, especially in regions where water conservation is critical.

## Conclusion

This study highlights the potential of organic amendments—biochar, compost, and sludge—to improve the water retention and hydraulic behavior of sandy soils. Results from a 441-day lysimeter experiment demonstrate that the effects observed for individual amendments may not simply accumulate when applied in combination. In fact, co-application often led to interactive effects that differed from those of single amendments alone. Among all treatments, the combination of biochar, compost, and sludge (treatment F) produced the most consistent improvements in soil water retention, reduced drainage, and stabilized soil water content across varying seasonal conditions.

Biochar was particularly effective in reducing drainage losses and enhancing soil actual evaporation, likely due to its high porosity and water retention capacity. Compost and sludge contributed organic matter and fine particles, improving soil structure and buffering soil water fluctuations. Regression analyses confirmed that single-point soil hydraulic properties (*K*_*s*_, FC, PAW) were well correlated with measured drainage amounts, indicating that such parameters are valuable tools for predicting amendment performance in sandy soils.

A limitation of this study is the exclusion of vegetation, which can influence evapotranspiration dynamics. Future research should evaluate amendment effects under cropping systems and assess long-term performance under real agricultural conditions.

Based on our findings, we recommend the use of integrated organic amendment strategies that combine biochar with compost and/or sludge to enhance soil water retention and reduce drainage in coarse-textured soils. Such strategies are particularly promising for sustainable agriculture in arid and semi-arid environments where water use efficiency is critical.

## Supplementary Information

Supplementary Material 1.

## Data Availability

The datasets generated and/or analyzed during the current study, as well as the materials used, are available from the corresponding author on reasonable request.
