# Supplementary material for: Impact of biochar, compost, and sludge amendments on the soil water balance of a sandy soil
Source: Biochar. 2026 Jan 19;8(1):14. doi: 10.1007/s42773-025-00509-4 (PMC12812768; doi:10.1007/s42773-025-00509-4)
Supplement: Supplementary file 1 — Supplementary Material 1. [file 42773_2025_509_MOESM1_ESM.docx]

**Impact of biochar and compost amendments on the soil water balance of a clayic sand soil**

**- SUPPLEMENTARY MATERIAL -**

Slaven Tenodi^a^, Snezana Maletic^a^, Marijana Kragulj Isakovski^a^, Jens Kruse^b^, and Lutz Weihermüller^b*^

*^a^ University of Novi Sad, Faculty of Sciences, Department of Chemistry, Biochemistry and Environmental Protection, Trg Dositeja Obradovica 3, 21000 Novi Sad, Serbia*

*^b^ Institute of Bio- and Geosciences - IBG-3, Agrosphere, Forschungszentrum Jülich GmbH, Jülich, Germany*

* Corresponding author: Lutz Weihermüller

Agrosphere Institute, Leo-Brandt-Strasse, 52425 Jülich, Germany, email. l.weihermueller@fz-juelich.de

**1. Retention and conductivity models for soil amendments**

The fitted retention and hydraulic conductivity functions shown for the six treatments (A to F) using the dual-porosity model of Durner (1994) highlight important differences in soil water retention and movement characteristics due to the various amendments.

The retention curves for all treatments exhibit a steep decline, reflecting the typical behavior of sandy soils with limited water-holding capacity. However, treatments containing biochar (A, D, E, and F) tend to show slightly higher retention at lower pressure heads compared to treatments without biochar (B and C). This suggests that biochar contributes to enhanced water retention by increasing the soil’s microporosity, allowing for more effective storage of plant-available water. Notably, treatments with combinations of amendments, such as D (biochar + sludge), E (biochar + compost), and F (biochar + sludge + compost), display smoother and broader retention curves, indicative of improved pore size distribution and water-holding capacity.


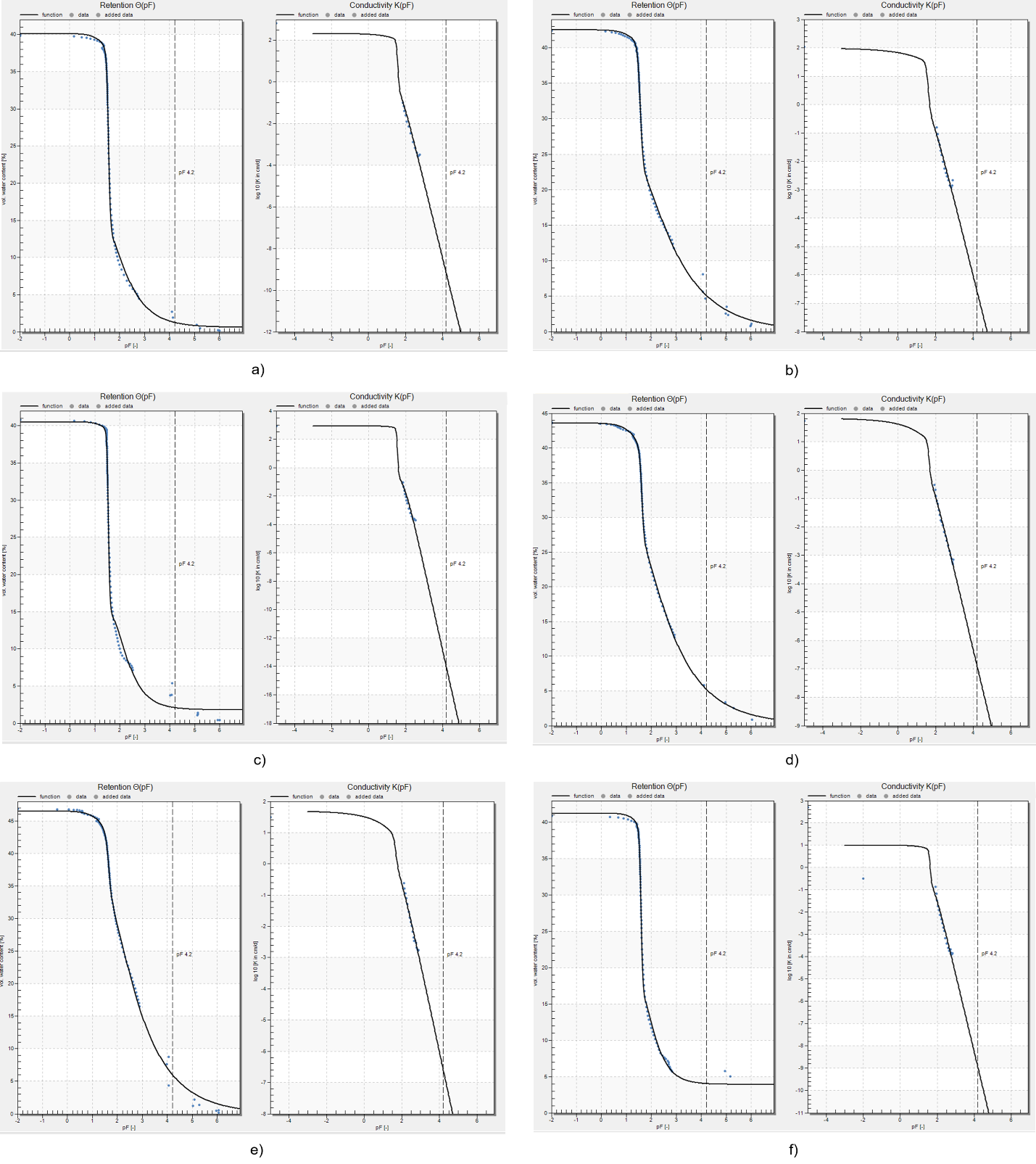


**Figure SM1.** Fitted retention and hydraulic conductivity functions using the dual-porosity model of Durner (1994) to the measured data for a) treatment A (sandy soil + biochar), b) treatment B (sandy soil + sludge), c) treatment C (sandy soil + compost), d) treatment D (sandy soil + biochar + sludge), e) treatment E (sandy soil + biochar + sludge + compost), and f) treatment F (sandy soil + biochar + compost).

The hydraulic conductivity functions reveal the effect of the amendments on water movement through the soil. Treatments with biochar, particularly when combined with compost (E) or sludge and compost (F), show reduced hydraulic conductivity at higher pressure heads. This suggests that biochar helps to slow down water percolation, promoting water availability in the root zone. Conversely, treatments with sludge (B) or compost alone (C) demonstrate higher conductivity at similar pressures, reflecting their limited ability to enhance water retention compared to biochar-inclusive treatments. The triple amendment (F) achieves the most balanced profile, with moderate hydraulic conductivity and enhanced water retention, indicating synergistic effects of combining biochar, sludge, and compost.

**2. Soil temperature**

Soil temperature was measured at 10 and 20 cm depths to monitor the thermal dynamics across the lysimeter treatments. Measurements are presented in Figures SM2, SM3 and SM4. Figures SM2 and SM3 offer a comprehensive view of soil temperature dynamics at two distinct depths (10 and 20 cm, measured from the bottom to the top of the lysimeters) across various amendments. These include biochar, sludge, compost, and their combinations. Figure SM4 further aggregates data across all treatments, emphasizing broader trends in soil temperature behavior.


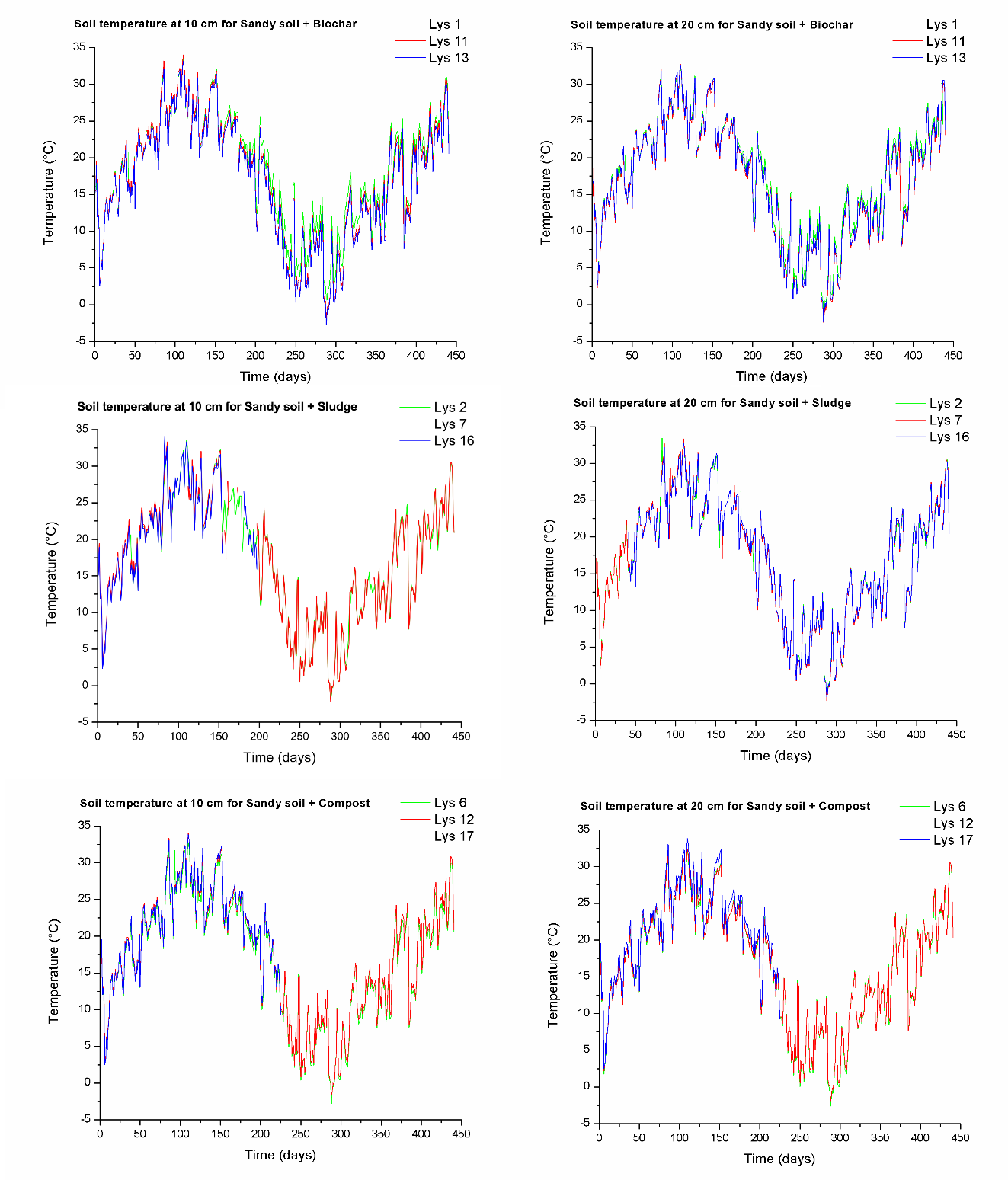


**Figure SM2.** Soil temperature (°C) over the experimental period of 441 days for different soil treatments: sandy soil + biochar (treatment A), sandy soil + Sludge (treatment B), and sandy soil + compost (treatment C), at two depths (10 and 20 cm from the bottom of the lysimeters) for the 3 replicated lysimeters. Days of the experiment were shown, whereby the start of the experiment was at 31^st^ of March 2023.


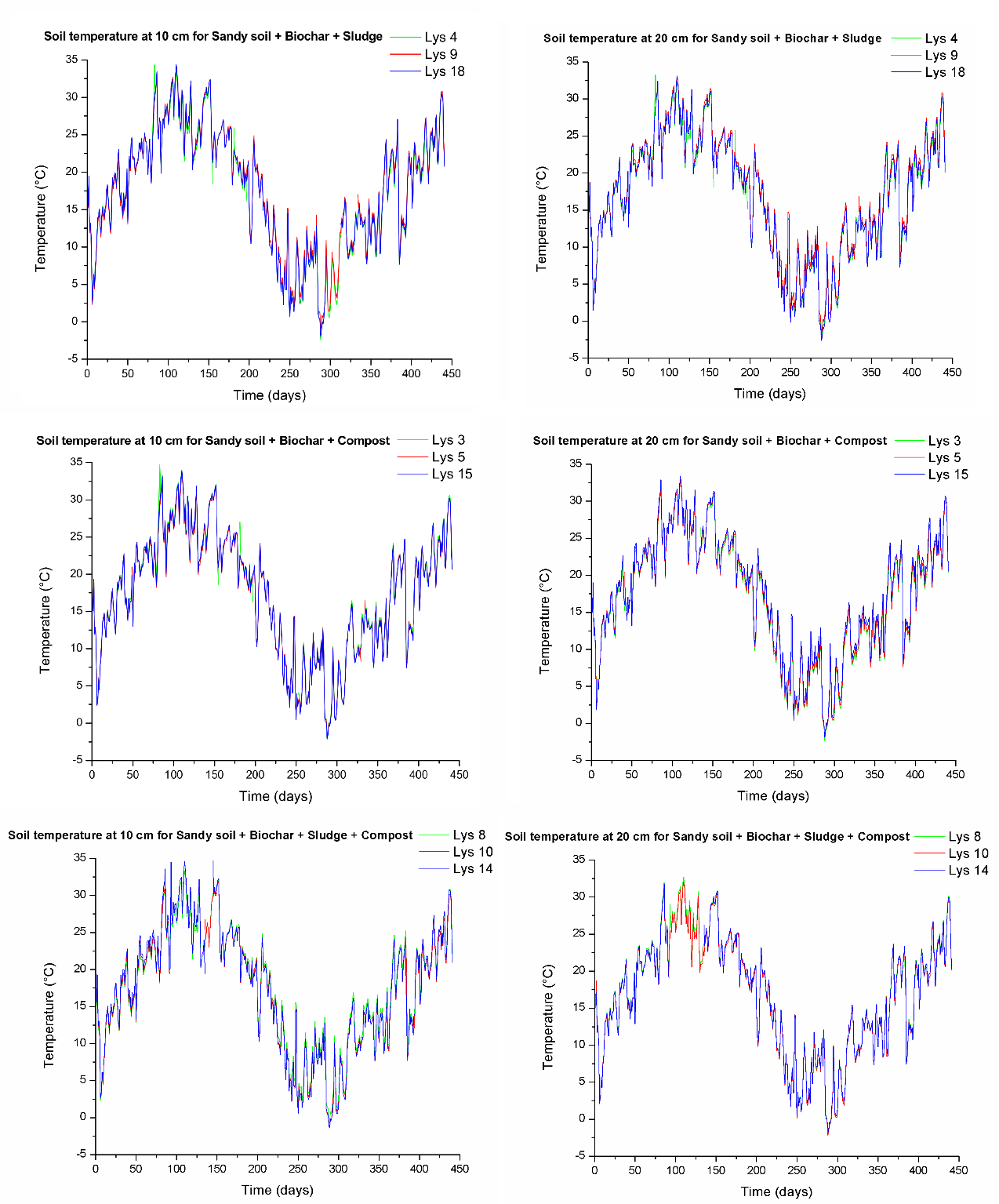


**Figure SM3.** Soil temperature (°C) over the experimental period of 441 days for different soil treatments: sandy soil + biochar + sludge (treatment D), sandy soil + biochar + compost (treatment E), and sandy soil + biochar + sludge + compost (treatment F), at two depths (10 and 20 cm from the bottom of the lysimeters) for the 3 replicated lysimeters. Days of the experiment were shown, whereby the start of the experiment was at 31^st^ of March 2023.

The seasonal patterns evident in all figures demonstrate natural fluctuations in soil temperature, with peaks during the summer and troughs during the winter. This is expected given the climatic conditions during the experiment and highlights the responsiveness of the experimental setup to ambient environmental variations. Depth-wise, the 10 cm measurements consistently exhibit greater fluctuations compared to the 20 cm measurements. This is likely due to the proximity of the 10 cm sensors to the soil surface, making them more susceptible to atmospheric temperature changes. In contrast, the 20 cm depth benefits from thermal buffering provided by the soil, which dampens rapid fluctuations.

A key observation across all figures is the lack of significant variation in soil temperature between treatments. Whether amended with biochar, sludge, compost, or their combinations, the temperature trends are remarkably similar. This suggests that the amendments primarily influence the soil's hydraulic properties rather than its thermal dynamics. The minimal impact of treatments on soil temperature across both depths reinforces the study’s focus on water retention and drainage performance.

Figure SM4 consolidates the temperature trends across all treatments, further underscoring the consistency in thermal behavior. This uniformity simplifies the interpretation of hydraulic data by confirming that temperature-related effects are not confounding the results.

Overall, the consistency in temperature dynamics across treatments and depths validates the experimental design and highlights that the primary influence of the amendments lies in altering the hydraulic properties of the soil. This insight is critical for drawing accurate conclusions about the amendments' effects on water retention and movement without interference from thermal variability.


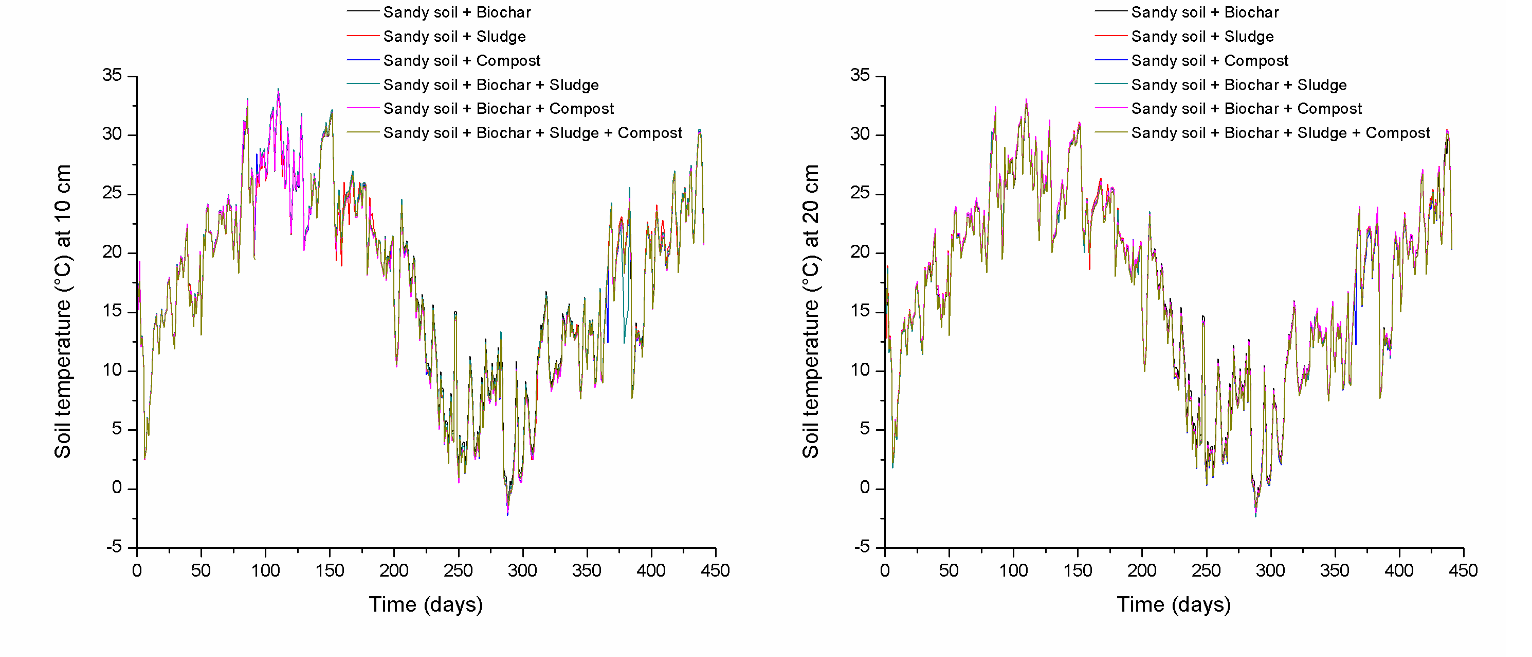


**Figure SM4.** Average values (*n* = 3 for soil treatments) of soil temperature (°C) over the experimental period of 441 days for different soil treatments: sandy soil + biochar (treatment A), sandy soil + sludge (treatment B), sandy soil + compost (treatment C),

sandy soil + biochar + sludge (treatment D), sandy soil + biochar + compost (treatment E), and sandy soil + biochar + sludge + compost (treatment F), at two depths (10 and 20 cm from the bottom of the lysimeters).

**2. Statistical analysis – ANOVA and Tukey HSD**

Table SM1 provides detailed results of the one-way ANOVA and Tukey HSD post-hoc analysis for cumulative drainage data across the six treatments (A-F). The ANOVA analysis showed a statistically significant effect of treatments on cumulative drainage (F = 13.182, *p* = 0.0002), demonstrating that the soil amendments significantly influence water movement through the soil profile. The Tukey HSD analysis revealed several key pairwise differences:

- **Treatment F vs. A:** The combination of biochar, sludge, and compost in treatment F significantly reduced cumulative drainage compared to biochar alone (treatment A), confirming the synergistic effect of combining amendments to retain water.
- **Treatment F vs. C:** Similarly, treatment F outperformed compost alone (treatment C) in reducing drainage, underscoring the limitations of compost as a standalone amendment for sandy soils.
- **Treatment C vs. D:** The combination of biochar and sludge in treatment D significantly outperformed compost alone (treatment C), highlighting the importance of incorporating biochar for effective water retention.

**Table SM1.** Results of one-way ANOVA and Tukey HSD post-hoc analysis for cumulative drainage data across treatments

| **One-way ANOVA results – Drainage data** | | | | | | |
| --- | --- | --- | --- | --- | --- | --- |
| Source of variation | SS | df | MS | F | *p*-value | F crit |
| Between groups | 40889.0 | 5 | 8177.8 | 13.182 | **0.0002** | 3.106 |
| Within groups | 7444.7 | 12 | 620.40 | - | - | - |
| Total | 48333.8 | 17 | - | - | - | - |
| **Tukey HSD results – Drainage data** | | | | | | |
| Group 1 | Group 2 | Mean diff | *p*-adjusted | Lower | Upper | Reject |
| **A** | **B** | **-90.154** | **0.0082** | **-158.465** | **-21.844** | **True** |
| A | C | 8.433 | 0.9980 | -59.878 | 76.743 | False |
| **A** | **D** | **-118.407** | **0.0009** | **-186.718** | **-50.096** | **True** |
| A | E | -54.727 | 0.1483 | -123.038 | 13.584 | False |
| **A** | **F** | **-92.040** | **0.0070** | **-160.354** | **-23.733** | **True** |
| **B** | **C** | **98.587** | **0.0041** | **30.276** | **166.898** | **True** |
| B | D | -28.253 | 0.7326 | -96.563 | 40.058 | False |
| B | E | 35.427 | 0.5324 | -32.883 | 103.738 | False |
| B | F | -1.889 | 1.0000 | -70.200 | 66.421 | False |
| **C** | **D** | **-126.839** | **0.0005** | **-195.150** | **-58.529** | **True** |
| C | E | -63.160 | 0.0762 | -131.470 | 5.151 | False |
| **C** | **F** | **-100.476** | **0.0036** | **-168.787** | **-32.166** | **True** |
| D | E | 63.680 | 0.0730 | -4.631 | 131.991 | False |
| D | F | 26.363 | 0.7820 | -41.947 | 94.674 | False |
| E | F | -37.317 | 0.4810 | -105.627 | 30.994 | False |

**Note:** Bolded values indicate statistically significant results (*p* < 0.05), including overall ANOVA tests and pairwise comparisons based on Tukey HSD.

These results underscore the superiority of combined amendments in mitigating water loss through drainage, with treatment F consistently demonstrating the lowest drainage values.

Table SM2 presents the analysis of storage data, which serves as a proxy for soil water content (SWC). The ANOVA results did not show statistically significant differences among treatments (F = 2.314, *p* = 0.1086), indicating high variability in SWC across replicates. However, the trends observed in the Tukey HSD analysis provide valuable insights into treatment effects.

**Table SM2.** Results of one-way ANOVA and Tukey HSD post-hoc analysis for cumulative storage data across treatments

| **One-way ANOVA results – Storage data** | | | | | | |
| --- | --- | --- | --- | --- | --- | --- |
| Source of variation | SS | df | MS | F | *p*-value | F crit |
| Between groups | 416.2 | 5 | 83.23 | 2.314 | 0.1084 | 3.106 |
| Within groups | 431.7 | 12 | 35.97 | - | - | - |
| Total | 847.9 | 17 | - | - | - | - |
| **Tukey HSD results – Storage data** | | | | | | |
| Group 1 | Group 2 | Mean diff | *p*-adjusted | Lower | Upper | Reject |
| A | B | -10.175 | 0.3584 | -26.624 | 6.274 | False |
| A | C | -0.329 | 1.0000 | -16.779 | 16.120 | False |
| A | D | -0.232 | 1.0000 | -16.681 | 16.217 | False |
| A | E | 0.552 | 1.0000 | -15.897 | 17.002 | False |
| A | F | -10.195 | 0.3565 | -26.645 | 6.254 | False |
| B | C | 9.846 | 0.3904 | -6.604 | 26.295 | False |
| B | D | 9.943 | 0.3808 | -6.506 | 26.392 | False |
| B | E | 10.727 | 0.3088 | -5.722 | 27.177 | False |
| B | F | -0.020 | 1.0000 | -16.470 | 16.429 | False |
| C | D | 0.097 | 1.0000 | -16.352 | 16.547 | False |
| C | E | 0.881 | 1.0000 | -15.568 | 17.331 | False |
| C | F | -9.866 | 0.3883 | -26.317 | 6.583 | False |
| D | E | 0.784 | 1.0000 | -15.665 | 17.234 | False |
| D | F | -9.963 | 0.3788 | -26.413 | 6.486 | False |
| E | F | -10.748 | 0.3070 | -27.197 | 5.702 | False |

Key observations from the Tukey HSD analysis include:

- **Non-significant differences:** Despite the lack of statistical significance, the mean differences suggest that treatments combining biochar with sludge or compost (e.g., treatments D, E, and F) tend to enhance SWC stability compared to individual amendments.
- **Variability among replicates:** The lack of significant differences likely stems from inherent variability within replicates, emphasizing the need for larger sample sizes or longer experimental periods to capture subtle treatment effects.

While not statistically significant, these findings align with broader observations in the manuscript that combined amendments improve soil water retention over time. Future studies should explore whether prolonged experimental durations or varying environmental conditions amplify these effects.

Table SM3 provides a comprehensive analysis of cumulative actual evaporation (E_act_) data. The ANOVA analysis revealed significant differences among treatments (F = 10.503, *p* = 0.0004), reflecting the impact of amendments on water loss to the atmosphere. Highlights from the Tukey HSD analysis include:

- **Treatment F vs. A and C:** Treatment F exhibited moderate evaporation rates, outperforming biochar alone (treatment A) and compost alone (treatment C) in balancing water retention with atmospheric loss. This highlights the importance of combining biochar with sludge and compost to stabilize evaporation.
- **Treatment C vs. D:** The significant difference between compost alone (treatment C) and the combination of biochar and sludge (treatment D) further emphasizes the role of biochar in enhancing soil hydraulic properties and reducing evaporation.
- **Balance in Treatment F:** The combination of all three amendments in treatment F maintained an optimal balance, minimizing water loss while retaining sufficient moisture for potential plant use.

These results reinforce the importance of integrating biochar with organic amendments to enhance soil water management, particularly in regions prone to high evaporation rates.

**Table SM3.** Results of one-way ANOVA and Tukey HSD post-hoc analysis for cumulative actual evaporation data across treatments

| **One-way ANOVA results – Actual evaporation data** | | | | | | |
| --- | --- | --- | --- | --- | --- | --- |
| Source of variation | SS | df | MS | F | *p*-value | F crit |
| Between groups | 37273.5 | 5 | 7454.7 | 10.503 | **0.0004** | 3.106 |
| Within groups | 8517.2 | 12 | 709.8 | - | - | - |
| Total | 45790.7 | 17 | - | - | - | - |
| **Tukey HSD results – Actual evaporation data** | | | | | | |
| Group 1 | Group 2 | Mean diff | *p*-adjusted | Lower | Upper | Reject |
| **A** | **B** | **79.979** | **0.0293** | **6.914** | **153.045** | **True** |
| A | C | -8.762 | 0.9983 | -81.827 | 64.304 | False |
| **A** | **D** | **118.175** | **0.0016** | **45.110** | **191.240** | **True** |
| A | E | 55.279 | 0.1866 | -17.786 | 128.345 | False |
| **A** | **F** | **81.848** | **0.0253** | **8.783** | **154.914** | **True** |
| **B** | **C** | **-88.741** | **0.0148** | **-161.807** | **-15.676** | **True** |
| B | D | 38.196 | 0.5246 | -34.870 | 111.261 | False |
| B | E | -24.700 | 0.8576 | -97.766 | 48.365 | False |
| B | F | 1.869 | 1.0000 | -71.196 | 74.935 | False |
| **C** | **D** | **126.937** | **0.0009** | **53.871** | **200.002** | **True** |
| C | E | 64.041 | 0.0992 | -9.024 | 137.106 | False |
| **C** | **F** | **90.610** | **0.0128** | **17.545** | **163.676** | **True** |
| D | E | -62.896 | 0.1080 | -135.961 | 10.170 | False |
| D | F | -36.327 | 0.5733 | -109.392 | 36.739 | False |
| E | F | 26.569 | 0.8188 | -46.496 | 99.635 | False |

**Note:** Bolded values indicate statistically significant results (*p* < 0.05), including overall ANOVA tests and pairwise comparisons based on Tukey HSD.
